# Unidirectional Current in Layered Metal Hexacyanometallate Thin Films: Implication for Alternative Wet-Processed Electronic Materials

**DOI:** 10.1021/acsomega.3c06447

**Published:** 2023-11-08

**Authors:** Lena Gerhards, Gunther Wittstock

**Affiliations:** School of Mathematics and Science, Institute of Chemistry, Carl von Ossietzky University of Oldenburg, 26111 Oldenburg, Germany

## Abstract

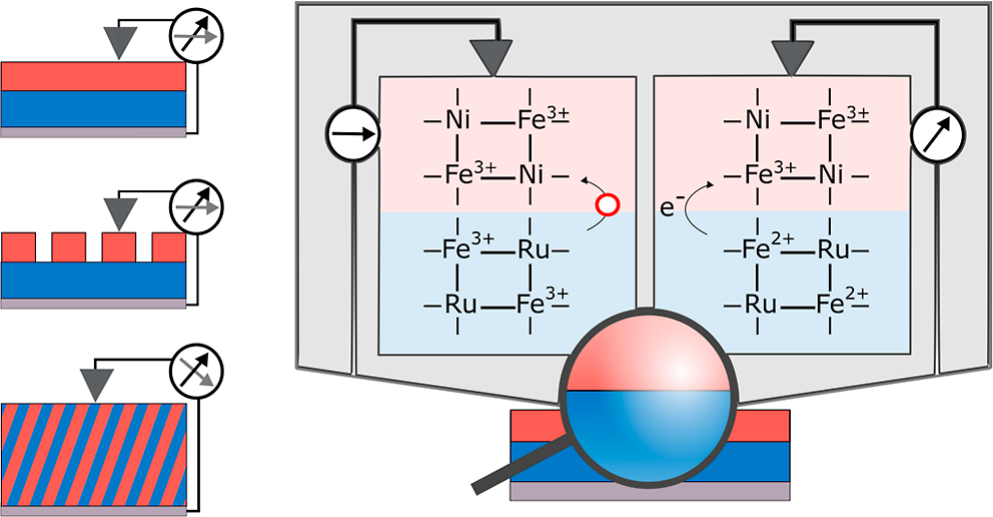

Rectifying behavior of alternative electronic materials is demonstrated with layered structures of a crystalline coordination network whose mixed ionic and electronic conductivity can be manipulated by switching the redox state of coordinated transition-metal ions. The coordinated transition-metal ions can convey additional functionality such as (redox)catalysis or electrochromism. In order to obtain rectifying behavior and charge trapping, layered films of such materials are explored. Specifically, layered films of iron hexacyanoruthenate (Fe-HCR) and nickel hexacyanoferrate (Ni-HCF) were formed by the combination of different deposition procedures. They comprise electrodeposition during voltammetric cycles for Fe-HCR and Ni-HCF, layer-by-layer deposition of Ni-HCF without redox chemistry, and drop casting of presynthesized Ni-HCF nanoparticles. The obtained materials were structurally characterized by X-ray diffraction analysis, X-ray photoelectron spectroscopy, scanning electron microscopy, transmission electron microscopy for nanoparticles, and scanning force microscopy (SFM). Voltammetry in 1 mol L^–1^ KCl and current–voltage curves (*I*–*V* curves) recorded between a conductive SFM tip and the back electrode outside of an electrolyte solution demonstrated charge trapping and rectifying behavior based on the different formal potentials of the redox centers in the films.

## Introduction

Alternative electronic materials that can be wet-processed and coated onto existing surfaces represent an area of intensive research.^1,2^ In this realm, coordination network compounds can offer additional functionalities due to the catalytic and light-absorbing properties of the coordinated transitional-metal cations.^3−6^ Significant progress has been reached in coating coordination network compounds in a structurally well-defined way on various, surface-modified substrates.^7,8^ The realization of basic electronic functions, especially in the dry state, can be challenging because such layered structures require the combination of two materials with compatibility in structure and chemical stability.

Metal hexacyanometallates M^1^-HCM^2^ (M^1^ = Fe, Ni, Cu, Co, Zn, etc.; HCM^2^ = HCF for hexacyanoferrates, HCR for hexacyanoruthenates) are coordination network compounds.^9,10^ The general stoichiometry can be described as A_*x*_M^1^_*y*_[M^2^(CN)_6_]_*z*_ × *n*H_2_O, where A stands for an alkali metal cation (e.g., K^+^ or Na^+^), M^1^ refers to the N-coordinated transition-metal ion, and M^2^ is the C-coordinated transition-metal ion.^9−11^ Unit cells of metal hexacyanometalates vary, depending on the preference of the contained transition-metal ions for octahedral or tetrahedral coordination. Fe_4_[Ru(CN)_6_] and KNi[Fe(CN)_6_] crystallize in face-centered cubic unit cells, which represents the most common case for metal hexacyanometallates.^12−14^ Here, both types of metal centers occur in an octahedral coordination^15^ interconnected via cyanide linkers (CN^–^).^16^ However, there are also exceptions from this prevailing trend (tetrahedral coordination in Zn_2_[Fe(CN)_6_] × 2H_2_O and Zn_3_K_2_[Fe(CN)_6_]_2_ × *x*H_2_O).^15^ In some cases, phase transitions have been observed upon oxidation/reduction and concomitant intercalation of cations (monoclinic crystal lattice in Na_2_Fe^II^[Fe^II^(CN)_6_] but cubic crystal lattice in NaFe^III^[Fe^II^(CN)_6_]).^17^ The large number of M^1^–M^2^ combination is the main reason for the diverse electronic, redox, photonic, and magnetic properties of M^1^-HCM^2^ compounds^18,19^ that have led to a range of application, e.g., for sensors,^18,20−22^ batteries,^23−25^ gas storage,^26−28^ catalysis,^29−32^ environmental remediation,^33−35^ and medical research.^36,37^ The redox properties are connected to valence changes of the transition-metal cations M^1^ and M^2^, and mixed-valence conditions are frequently encountered.^38^

M^1^-HCM^2^s can be synthesized by different synthesis routes.^10,18,39^ For instance, (electro)chemical syntheses can be used to induce a valence change in the dissolved precursor close to the electrode surface. The change in valence state causes a decrease of the solubility product and may lead to precipitation of M^1^-HCM^2^ materials directly on the electrode surface.^40^ There are also other film preparation methods (e.g., layer-by-layer (LbL) deposition^41,42^ and vapor-assisted conversion^41^) that result in films directly on a substrate. Precipitation methods may yield nanoparticles^43^ or powders.^39,44^

Films were also prepared with combinations of different M^1^-HCM^2^ materials. Tan and co-workers^45^ studied films containing Cu_*x*_Ni_*y*_-HCF with different stoichiometries of Cu and Ni, which was easily tuned by the reactant molar ratio. Electrochemical responses of Cu_*x*_Ni_*y*_-HCF show the redox features of the Ni-HCF and Cu-HCF films. The intensity of the respective voltammetric signals depends on the amount of Ni or Cu contained in the material. The cycling stability improved with increase in the Ni content.

Different M^1^-HCM^2^ materials can also be combined as stratified layers. Such a layered system has been used to modify electrodes so that they can sustain only a unidirectional current flow.^46,47^ In agreement with the earlier literature, especially ref (46), we refer to “charge trapping”, when the outer layer of a layered sample in contact with an electrolyte solution retains its redox state even if the support electrode has reached a potential at which a redox reaction of the outer layer should occur. We use the term “rectifying behavior” for characteristics of current–voltage curves of the layered sample when it is in contact with two metallic conductors outside of an electrolyte solution. Both phenomena are based on the same internal processes at the interface between the two material layers.

Kulesza and co-workers^46^ combined Fe-HCF as an inner layer and Ni-HCF as an outer layer separated by a matrix of intrinsically conducting poly(*N*-methylpyrrole). The matrix avoided direct contact of the outer layer to the electrode surface and also direct contact of the two M^1^-HCM^2^ layers that could lead to the formation of a surface layer, in which transition-metal ions may be exchanged in the HCF lattices of one or both layers. The system was prepared as (i) a modified electrode and studied in contact with a liquid electrolyte and (ii) as-layered thick films obtained by pressed powders that were studied in the solid state without contact to a liquid electrolyte in a two-electrode arrangement. This system showed charge trapping in cyclic voltammetry (CV) and rectifying behavior in dry solids.^46^

Ono et al.^47^ studied the same system but used spin-coating for deposition of the outer layer on the inner layer and analyzed the charge trapping behavior by means of CV.

Karyakin and co-workers^48^ investigated the bilayer system of Fe-HCF and Ni-HCF in order to improve the material as an electrocatalyst for H_2_O_2_ reduction. The system was obtained by electrodeposition of Fe-HCF first, followed by another electrodeposition step for Ni-HCF directly on the first layer. The bilayer film retained its catalytic activity at a level similar to that of the initial activity of an Fe-HCF film but showed better stability. Interestingly, no charge trapping was observed at the bilayer system of Fe-HCF as the inner layer and Ni-HCF as the outer layer. This suggests that the use of sequential electrodeposition of both layers without a polymer as the separator is not a successful way to produce rectifiers. In a more general sense, the result highlights the importance of details in the deposition techniques on the functional properties of multilayer materials.

According to Abruña et al.,^49^ rectifying behavior in the layered films of redox-active materials can be observed if (i) the inner layer insulates the outer layer from the electrode at all potential except for those where the inner layer is redox-active and can act as a mediator for the outer layer; (ii) the outer layer is permeable to the flow of counterions required to balance the charge in the inner layer after a redox transition; (iii) there should be an as low as possible energetic overlap between the redox levels of the inner and outer layer; and (iv) the outer layer can release the trapped charge to facilitate the repetitive rectification events.

In principle, there is a large combination of M^1^-HCM^2^ materials that could satisfy this condition, and some have already been presented but made use of polymeric binders to ensure the stratification of the layers.^46,49^ We aimed for a system that works without binders, in which the layers can be spectroscopically addressed by an element that occurs only in one of the two layers. Avoidance of organic binders is also of interest in electrocatalytic systems, in which they may be susceptible to degradation when in contact with redox-active transition-metal ions. This requires that the layers remain stable during prolonged cycling, and especially the inner layer should be prepared as a continuous film that—despite its nanoscopic thickness—prevents direct contact of the outer film to the substrate electrode via defects such as pinholes or cracks. On the other hand, the outer layer must grow as a film in direct contact to the inner layer, which may impose further restrictions on the structural compatibility of the materials.

**Figure 1:**
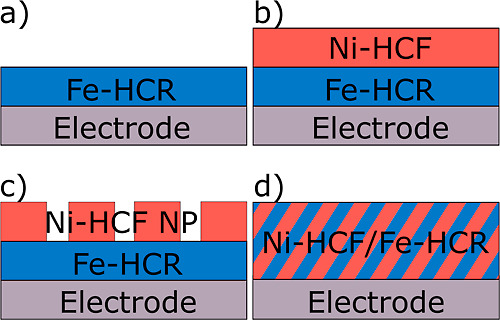
Schematic illustration of samples in this work. (a) Fe-HCR as thin film on a conductive electrode; (b) layered system of Fe-HCR and Ni-HCF; (c) layered system of Fe-HCR as a thin film and Ni-HCF nanoparticles; and (d) mixed system of Fe-HCR and Ni-HCF. Color code: blue, Fe-HCR; and red, Ni-HCF.

In this article, we specifically address the role of interfaces between different M^1^-HCM^2^ systems and aim to extend the range of suitable M^1^-HCM^2^ combinations that show charge trapping/rectifying behavior. To this end, we combined Fe-HCR as the inner layer and Ni-HCF as the outer layer produced by different deposition methods (Figure 1).

Fe-HCR has a repeating unit cell of Fe_4_^III^[Ru^II^(CN)_6_]_3_ or KFe^III^[Ru^II^(CN)_6_].^50^ This material was deposited electrochemically (Figure 1a).^50^ Ni-HCF was deposited by LbL deposition on an FeHCR film (Figure 1b) or by drop-casting of bulk-synthesized nanoparticle suspensions (Figure 1c). The stratified layer systems are compared to films, in which Fe-HCR and Ni-HCF were deposited by alternatingly conducting just one potential cycle in the respective precursor solutions (Figure 1d). For brevity, the film in Figure 1d is called mixed material and exhibited a simple superposition of signals of Fe-HCR and of Ni-HCF. In contrast, charge trapping was observed for the layered systems in Figure 1b,c in thin-film CV as well as rectifying behavior in current–voltage curves in the solid state.

## Experimental Section

### Materials

K_3_[Fe(CN)_6_] (≥99%, Alfa Aesar, Massachusetts, USA), NiCl_2_ × 6H_2_O (99.9%, metal trace basis, Sigma-Aldrich, Missouri, USA), K_4_[Ru(CN_6_)] × *x* H_2_O (Sigma-Aldrich), FeCl_3_ × 6H_2_O (99+%, Acros-Organics B. V. B. A., Thermo Fisher Scientific Inc., Waltham, Massachusetts, USA), and KCl (≥99.5% p.a., ACS, ISO, Carl Roth GmbH & Co. KG, Karlsruhe, Germany) were used. 4′-Mercapto-[1,1′-biphenyl]-4-carbonitrile and ethanol (Sigma-Aldrich) were used for monolayer preparation. All chemicals were used as received.

Indium tin oxide (ITO, 15–25 Ω/sq., Sigma-Aldrich), Au wire (99.999%, Goodfellow, Cambridge Ltd., Huntingdon, UK), Cr wire (99.7%, Goodfellow Cambridge Ltd.), and microscope glass slides (Thermo Fisher Scientific Inc.) were cleaned as specified below.

Deionized water was used for all solutions and cleaning procedures and was obtained from a PureLab Classic system (Elga LabWater, Germany, resistivity ≥ 18.2 MΩ cm).

### Preparation of Material

Au surfaces for the X-ray photoelectron spectroscopy (XPS) measurements were prepared by depositing 0.5 nm Cr and 150 nm Au onto cleaned microscope glass slides (sonication in ethanol for 5 min, water for 5 min, and UV/O_3_ cleaning for 10 min (UV TipCleaner, UV.TC.EU.003, Bioforce Nanosciences, Inc. Ames, IA, USA), drying in an Ar stream) using an evaporation chamber Tectra mini-coater (Tectra GmbH, Frankfurt am Main, Germany), which allows us to measure the thickness of the deposited layers by means of a quartz crystal balance (EM-Tec 6 MHz, gold electrode quartz crystals for thickness monitor, Micro to Nano, Haarlem, Netherlands). After Au deposition, the Au substrates were cleaned with acetone, ethanol, and water and dried in Ar stream. Afterward, the samples were cleaned by UV/O_3_ treatment for 10 min.

The films for voltammetric analysis were prepared on ITO. ITO was cleaned by acetone, ethanol, and water in a sonication bath for 5 min each. Subsequently, the substrates were dried in an Ar stream. Fe-HCR was electrochemically deposited during 15 potential cycles in the potential window of −0.2–0.6 V with a scan rate *v* = 40 mV s^–1^ in an aqueous solution of 1 mM K_4_[Ru(CN)_6_] + 1 mM FeCl_3_ + 70 mM KCl (Figure S1a). Ni-HCF (for Figure S1b) was deposited during 15 potential cycles at *v* = 40 mV s^–1^ between −0.0–0.75 V in 1 mmol L^–1^ NiCl_2_ + 0.5 mmol L^–1^ K_3_[Fe(CN)_6_] + 500 mmol L^–1^ KCl (Supporting Information 1).

Ni-HCF nanoparticles were synthesized according to the procedure of Li et al.^43^ Briefly, a 100 mL aqueous solution of 80 mmol L^–1^ NiCl_2_ and an equal volume of 73.5 mmol L^–1^ K_3_[Fe(CN)_6_] were mixed by simultaneously dropwise addition (10 mL h^–1^ of each solution) to 200 mL of deionized water. After complete addition, the solution was stirred for 18 h. The nanoparticles were centrifuged four times for 15 min at 4200 rpm (Megafuge 16, Thermo Scientific, USA) and stored in aqueous solution in a fridge at 4 °C for further use.

**Figure 2:**
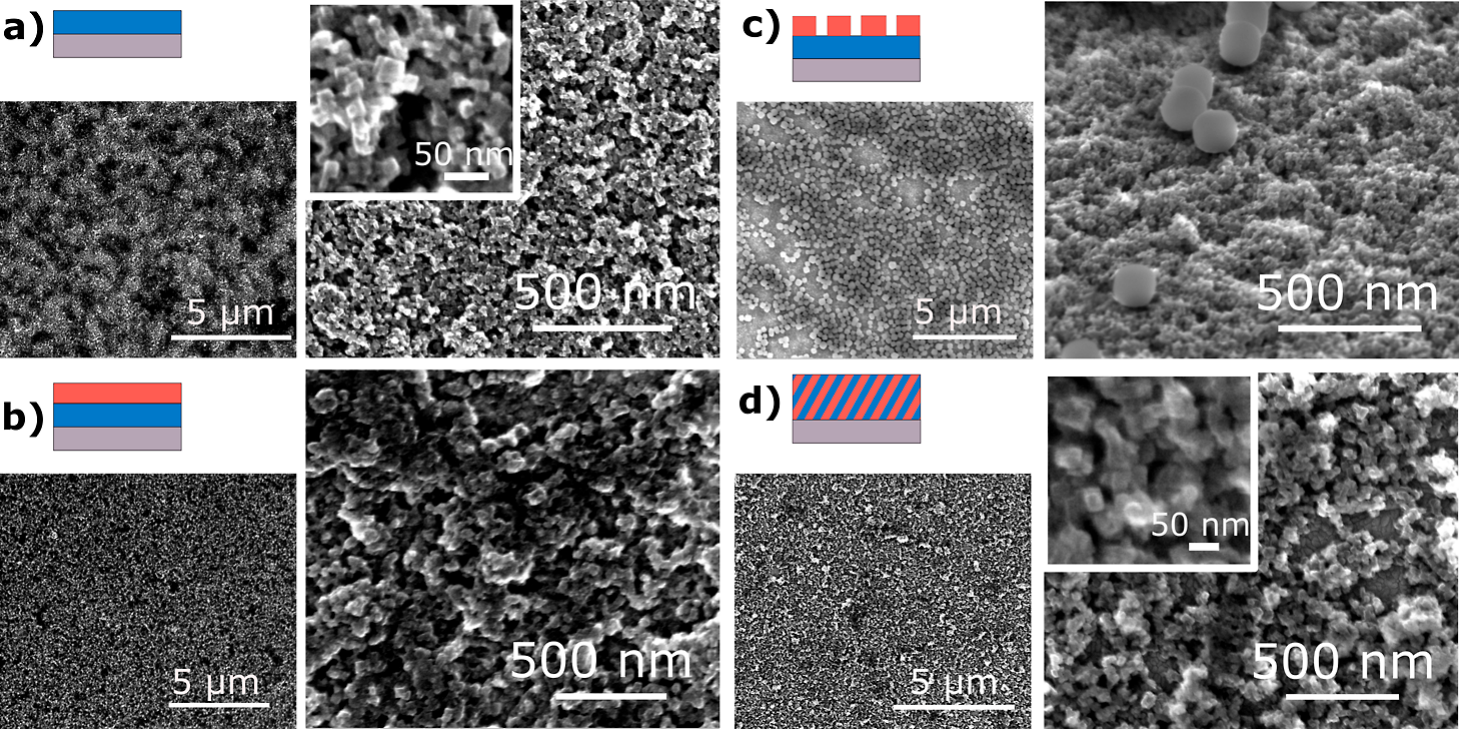
SEM of metal hexacyanometallate layers. (a) Fe-HCR, (b) Fe-HCR|Ni-HCF, (c) Fe-HCR|Ni-HCF nanoparticles, and (d) mixed material from alternating deposition of Fe-HCR and Ni-HCF thin films.

**Figure 3:**
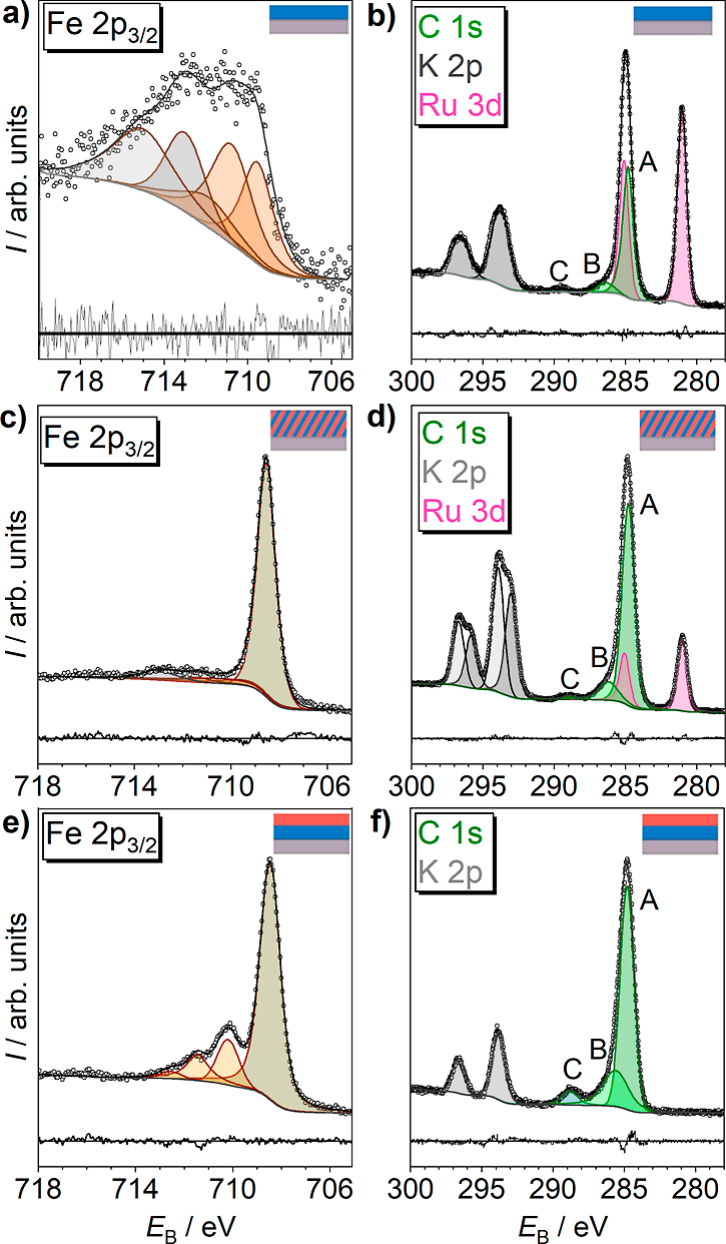
Fitting of the high-resolution Fe 2p_3/2_, C 1s, K 2p, and Ru 3d XP spectra. (a,b) Fe-HCR; (c,d) mixed material; and (e,f) layered material.

**Figure 4:**
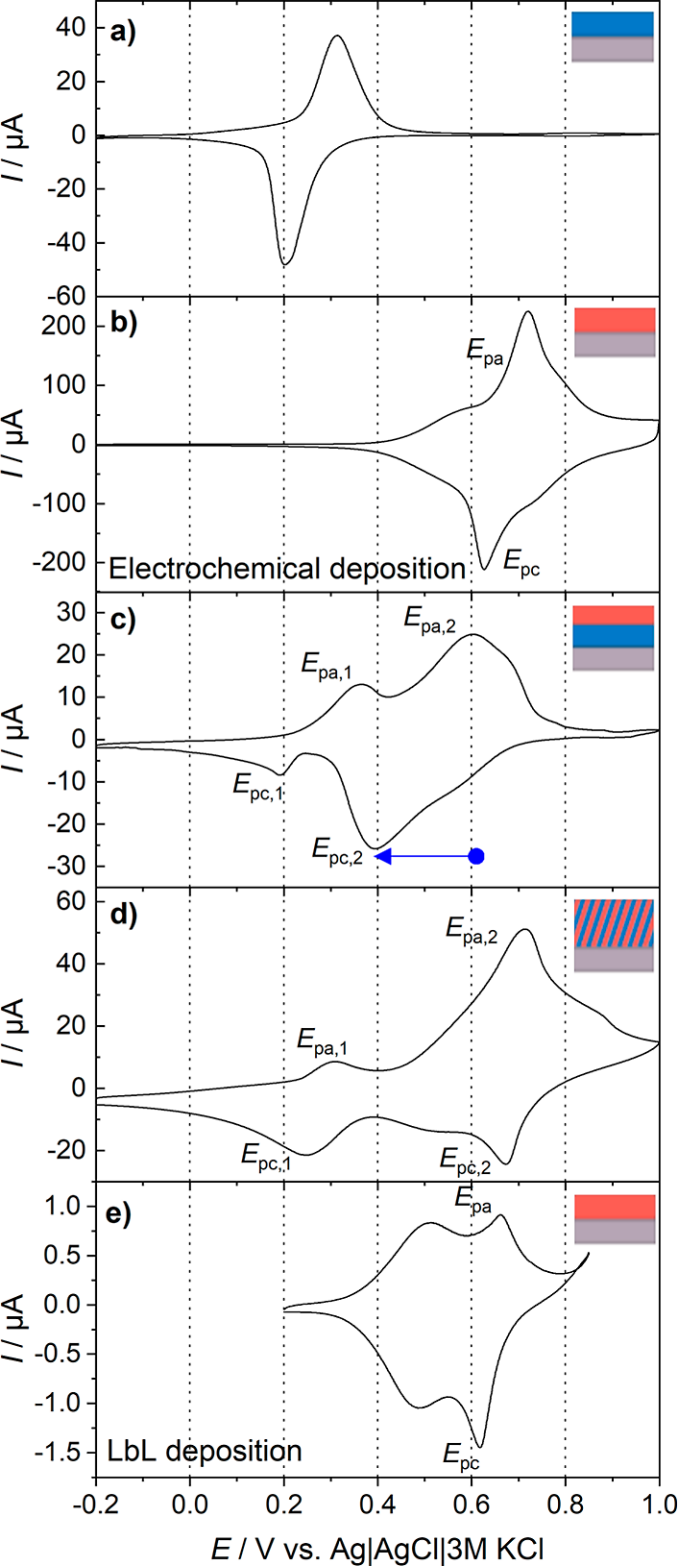
Cyclic voltammograms of (a) electrodeposited Fe-HCR; (b) electrodeposited Ni-HCF; (c) layered material with Fe-HCR as the inner layer and Ni-HCF as the outer layer; (d) mixed material of Fe-HCR and Ni-HCF; and (e) Ni-HCF obtained by LbL deposition of a nitrile-terminated Au surface. Voltammograms were recorded in 1 mol L^–1^ KCl at *v* = 0.01 V s^–1^. The electrode area is different in panels (a–e).

**Figure 5:**
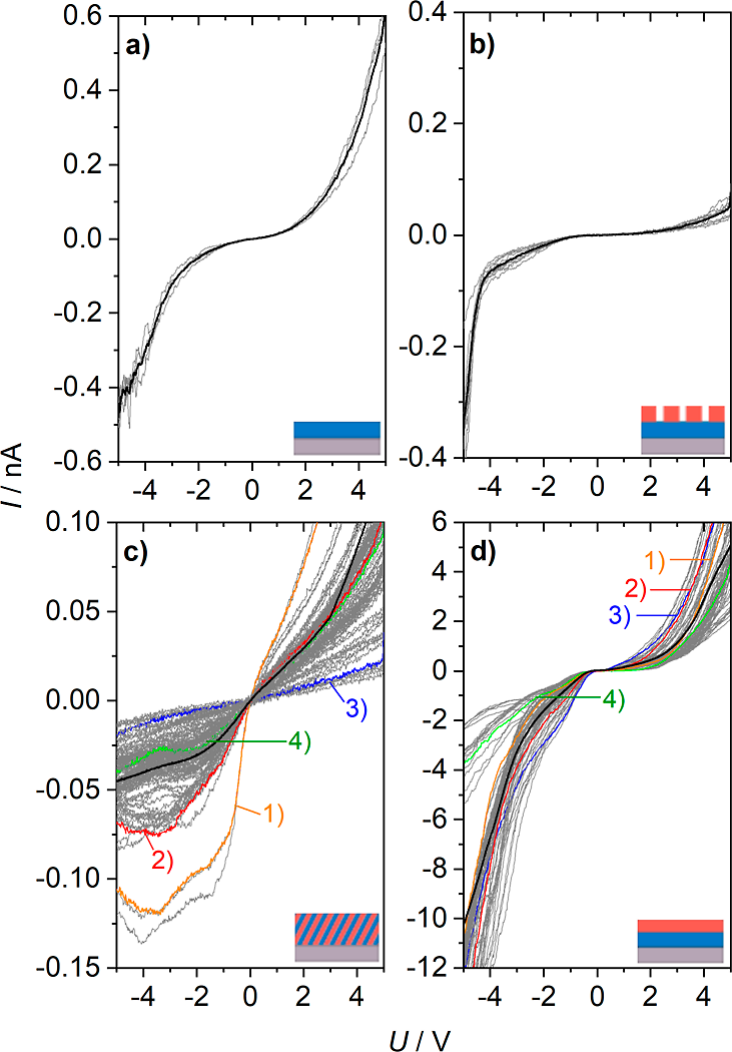
*I*–*V* curves of the metal hexacyanometallates. (a) Fe-HCR, (b) Ni-HCF nanoparticles on a Fe-HCR film, (c) mixed material, and (d) layered material Fe-HCR|Ni-HCF. For each material, several *I*–*V* curves were recorded at the same sample at different locations (see Supporting Information 6). They are shown in gray. The average of the curves for each sample is indicated by a black line. The colored curves 1–4 in panels (c,d) are selected curves that are shown with expanded scale in Figure S9 for (c) and Figure S10 for (d).

Ni-HCF-layered materials were obtained by LbL deposition^42^ from aqueous 20 mmol L^–1^ NiCl_2_ and aqueous 20 mmol L^–1^ K_3_[Fe(CN)_6_] solutions. The samples were exposed to each solution for 20 min. The number of dipping cycles and thus the film thickness were adjusted depending on the experiment; 14 cycles for Figure 2b, left; 2 cycles for Figure 2b, right; 18 cycles for Figures 3 and 5, S6, and S7b; and 4 cycles for Figure 4.

For the control experiments in Figure 4e, a Ni-HCF film was prepared directly on an Au surface that was coated by a self-assembled monolayer of 4′-mercapto-[1,1′-biphenyl]-4-carbonitrile. The substrate was obtained by exposing freshly evaporated Au substrate to a 10 mmol L^–1^ solution of the thiol in ethanol under an Ar atmosphere in the dark for 24 h. The samples were rinsed with ethanol and dried in an Ar stream.

The mixed material was obtained by an alternatingly executing one potential cycle at *v* = 40 mV s^–1^ between −0.2 and 0.6 V in aqueous 1 mmol L^–1^ K_4_[Ru(CN)_6_] + 1 mmol L^–1^ FeCl_3_ + 70 mmol L^–1^ KCl or between 0.0 and 0.75 V in 1 mmol L^–1^ NiCl_2_ + 0.5 mmol L^–1^ K_3_[Fe(CN)_6_] + 500 mmol L^–1^ KCl (Figure S1c). In total, seven cycles in each of the two solutions were executed. After each electrodeposition step, the samples were rinsed for 30 s with deionized water to remove noncoordinated species from the surfaces.

For comparison, drop-cast films were prepared from Ni-HCF nanoparticles by applying 10 μL of 0.28 mol L^–1^ (based on Ni^2+^ concentrations) nanoparticle solution.

### Characterization Methods

The voltammograms for film characterization were recorded with a CH660A potentiostat (CH Instruments Inc., Austin, USA) at 295 K in 1 mol L^–1^ KCl. The electrolyte solution was purged with Ar for 30 min before the measurement in a three-electrode setup consisting of the film-modified ITO electrode as the working electrode, a Pt foil as the auxiliary electrode, and a Ag|AgCl|3 mol L^–1^ KCl as the reference electrode (CH Instruments Inc.). All potentials are referred to the used Ag|AgCl|3 mol L^–1^ KCl as the reference electrode.

A droplet cell (Sensolytics, Bochum, Germany) was used for characterization of the layered material by CV. As a working electrode, the film-modified ITO electrode was used. A Pt wire (diameter 0.25 mm, Goodfellow) served as the auxiliary electrode. An chloridized Ag-wire (diameter 0.25 mm, Goodfellow) served as the pseudoreference electrode in this experiment. Potential differences relative to Ag|AgCl|3 M KCl were continuously checked and corrected accordingly.

Scanning force microscopy (SFM, Nanoscope IIIA controller with an Dimension 3100 stage, Nanoscope Software V5.3r3s3, Veeco Instruments Inc., Santa Barbara, CA, USA) was carried out in the tapping mode with NCHV-A cantilevers (42 N m^–1^, *f*_0_ = 320 kHz, Bruker, Camarillo, CA, USA). Current–voltage (*I*–*V*) curves in air were measured by using conductive SFM with conductive doped diamond tips (CDT-FMR, Nano and More, Wetzlar, Germany). More details are provided in Supporting Information 6.

Scanning electron microscopy (SEM) images were obtained by using a Helios Nanolab 600i system (FEI Company, Hillsboro, USA). A conducting connection between the upper conductive surface of the Au substrate and the SEM sample holder was made with the adhesive carbon tape.

XPS was performed using an ESCALAB 250 Xi instrument (Thermo Fisher Scientific, East Grinstead, UK) with monochromatized Al Kα radiation (*hv* = 1486.6 eV) and a spot size of 500 μm. The surveys spectra were measured with a pass energy of 200 eV and the individual lines with a pass energy of 10 eV. The samples were M^1^-HCM^2^ films on Au film electrodes. The carbon tape was used to electrically connect the sample to the sample holder. All spectra were referenced to the C 1s line at the binding energy *E*_B_ = 284.8 eV. Software Avantage v 5.932 (Thermo Fisher) was used for XPS curve fitting applying a “Smart Background” option (modified Shirley background) and using convolution of Gaussian and Lorentzian peak shapes.

Details of the powder X-ray diffraction experiments are listed in Supporting Information 3.

## Results and Discussion

For the following experiments, we compare the electrochemical and electrical behavior of samples with a stratified layer system schematically depicted in Figure 1b,c to (i) a Fe-HCR film (Figure 1a) and (ii) a mixed film in which both films were deposited alternatingly in an electrochemical LbL procedure (Figure 1d). The cyclic voltammograms of the film preparation for the samples depicted in Figure 1a–d are schematically shown in Figure S1a,c,d, respectively. It is very difficult to deposit an absolutely defect-free layered system. Defects in the first layer may greatly influence the electrochemical and electrical behavior, especially if the outer layer has a local contact to the substrate. The layered system was also realized as an ensemble of Ni-HCF nanoparticles on a continuous Fe-HCR film (Figure 1c) in order to compare *I*–*V* curves on the layered system with those on Fe-HCR on one and the same sample.

### Structural Characterization

The obtained layers show a crystalline structure as evident from the XRD pattern in Figure S6 expected for Fe-HCR and Ni-HCF.^13,14^ The SEM images in Figure 2a,b show continuous films of Fe-HCR and the layered material on the ITO surface. The cubic crystal morphology in the Fe-HCR film can be discerned in the inset of the zoomed image in Figure 2a. In Figure 2b, the zoomed image reveals a flaky structure; cubes cannot be identified. This fact may be caused by the LbL deposition process of Ni-HCF since the substrate on which the material is deposited can play an important role in morphology and therefore topography of the material. The Fe-HCR film, coated with Ni-HCF nanoparticles, is shown in Figure 2c. In the mixed material (Figure 2d), cubic particles can be identified as in the Fe-HCR film in Figure 2a. Beside this, the film does not cover the substrate completely.

The stratified layer material has a roughness of 21 nm [determined as root-mean-square *R*_q_ from scanning force microscopy (SFM) images on an area of 10 × 10 μm with a tip radius of 8 nm, Supporting Information 4]. The mixed film has an *R*_q_ of 45 nm (Supporting Information 4). The thickness of the Fe-HCR layer was around 80 nm. Taking this value and the experimental accessible thickness of the layered system of approximately 190 nm (Table S10), the thickness of Ni-HCF in the layered material amounts to around 110 nm. The thickness of the film with the mixed material was around 240 nm.

Transmission electron microscopy (TEM) images of the Ni-HCF nanoparticles and their size distribution are shown in Supporting Information 5. The nanoparticles have a cubic shape with an edge length between 170 and 300 nm (Figure S8). These particles can also be identified by their shape on the deposited Fe-HCR film, as shown in Figure 2c.

### Analysis of Valence States by XPS

The valence state of the transition-metal ions in the material was analyzed by XPS (Figure 3). The Fe 2p spectrum in Figure 3a shows the multiplet splitting expected for high-spin N-coordinated Fe^2+^ species in agreement with the expected structure for Fe-HCR.^51,52^ The approach of fitting the Fe 2p spectra using the calculated line pattern according to ref (53) is detailed in Supporting Information 2.1. The Fe 2p spectra of the mixed and layered materials in Figure 3c and e additionally show the emission from C-coordinated low-spin Fe^2+^ in Ni-HCF at *E*_B_ = 708.5 eV.

Ru 3d_5/2_ signals can be found in Figure 3d at 281.0 eV (Ru 3d_3/2_ at 285.08 eV overlapping with C 1s) and in Figure 3b at 281.03 eV (Ru 3d_3/2_ at 285.10 eV). The Ru 3d_5/2_ XP signal from K_4_[Ru(CN)_6_] × *x*H_2_O powder is detected at 281.01 eV; within the accuracy of the method all these binding energies agree with each other and with the reported value of 280.9 eV.^54^ The Ru 2p signal can be used as an indicator of whether the outer layer in the sample with stratified layers provides a complete coverage of the inner layer. The absence of Ru 3d_5/2_ at *E*_B_ = 281.0 eV in Figure 3f indicates that the outer Ni-HCF film (ca. 110 nm) is thicker than the information depth of XPS and does not contain significant pinholes.^55^

K 2p_3/2_/K 2p_1/2_ photoemission doublet is found at binding energies of 293.0 and 293.9 eV in Figure 3b,d,f. Interestingly, two doublets are discernible in Figure 3d for the mixed material indicative of K^+^ ions with different chemical environments. Comparison of peak width may also suggest more than one chemical state of K in Fe-HCR (Figure 3b vs 3f). Reasons for different chemical states can be interactions with the coordination network or different degrees of hydration. A detailed discussion is provided in Supporting Information 2.2.

The C 1s spectra (Figure 3b,d,f; signals A, B, and C for π → π* shakeup process in the cyanide ligand) are not considered for the analysis of the material since signals originating from CN^–^ cannot safely be distinguished from signals of adventitious carbon contamination present on all samples handled under ambient conditions.

### Electrochemical Behavior

Electrochemical deposition of Fe-HCR and Ni-HCF using CV indicates an incremental enhancement of the current and, thus, an increase in film thickness with every potential cycle (Figure S1). After completion of the film preparation, the modified electrodes were transferred to 1 mol L^–1^ KCl (without precursors for film formation). Typical redox responses of the deposited films at a slow scan rate of 10 mV s^–1^ are shown in Figure 4a,b for Fe-HCR and Ni-HCF, respectively. The formal potentials were estimated as 0.26 V for Fe-HCR (Figure 4a) and 0.68 V for Ni-HCF (Figure 4b) using the mean value of the anodic and cathodic peak potentials. All details are given in Tables S1–S4. In case of Ni-HCF, the main redox features of the film (Figure 4b, *E*_pa_, *E*_pc_) were used for the calculation since the voltammogram of Ni-HCF has two redox-pairs, which can be attributed to different stoichiometric forms.^56^Figure 4a,b show that the redox potentials of both materials are well separated from each other, which mainly depends on the transition metals that are N- or C-coordinated.^38^

The following simplified equations represent the redox reactions in Fe-HCR^50^ (1) and Ni-HCF^14^ (2)
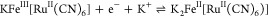
1
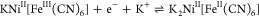
2The equations are simplified because they do not show vacancies and associated stoichiometry variation, place exchanges, and water content. All of these may vary with the particular preparation method.

Ni-HCF and other M^1^-HCM^2^ compounds can be considered as a solid solution of a form rich in alkali ions (also referred to as “soluble form”) and a structure containing vacancies on the M^2^ site (also called “insoluble form”).^46^ These different forms may give rise to discernible voltammetric signals. For Ni-HCF, the redox peaks at more positive potentials in Figure 4b,e are attributed to the K^+^-rich form [eq 2], and the peak pair at more negative peak potentials is assigned to the vacancy-containing form [eq 3].^56,57^
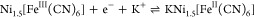
3It is noteworthy that variations in the deposition process may yield fractions of both forms in the resulting material, and thus the appearance of voltammograms may vary. Further small signal contribution may result from redox centers at the surface with a different coordination environment than in the bulk.

The occurrence of different forms of Ni-HCF is also evident from spectroelectrochemical experiments using polarization modulation infrared reflection absorption spectroscopy (PM IRRAS) detailed in Supporting Information 7, especially in Figure S14. Clear shifts of the *v*(C≡N) absorption mode are found also for Fe-HCR during oxidation–reduction cycles Figure S12. Unfortunately, there is considerable spectral overlap of the *v*(C≡N) modes for oxidized Fe-HCR and reduced Ni-HCF that hampers further detailed insights (Figure S13).

Figure 4c shows the voltammogram of the layered material on ITO with Fe-HCR as the inner layer and Ni-HCF as the outer layer. The peaks are located at *E*_pa,1_ = 0.36 V, *E*_pa,2_ = 0.60 V, *E*_pc,1_ = 0.19 V, and *E*_pc,2_ = 0.39 V. The redox processes leading to *E*_pa,1_ and *E*_pc,1_ in Figure 4c are not shifted significantly against the redox processes in the pure Fe-HCR in Figure 4a. This yields an estimation of the formal potential of Fe-HCR at 0.28 V (from *E*_pa,1_, *E*_pc,1_), which is only slightly more positive than that of the pure Fe-HCR film (0.26 V). However, the reduction peak *E*_pc,2_ corresponding to the reaction Ni–N≡C–Fe^3+^ → Ni–N≡C–Fe^2+^ in the layered material (Figure 4c) is shifted toward less positive potentials by 0.24 V compared to the pure Ni-HCF film (Figure 4b). This shift was expected for the layered material. The inner Fe-HCR layer mediates electron transfer between the electrode and the outer layer. The reaction at the interface between both materials can be described in a simplified way by reaction 5.
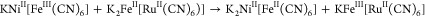
5As a control experiment, we investigated the CV of a Ni-HCF film directly deposited on a nitrile-terminated SAM-coated gold electrode (Figure 4e). The SAM is necessary to initiate the growth in the LbL procedure. Indeed, the signals in Figure 4e are shifted to less positive potentials than those in the voltammetry of the electrodeposited Ni-HCF film in Figure 4b. This can be due to different local structures in the Ni-HCF obtained by different deposition methods. However, the shift of the signals in the LbL-deposited film vs the electrodeposited film cannot account for the shift observed in the layered material.

The oxidation reaction of K_2_Ni^II^[Fe^II^(CN)_6_] to KNi^II^[Fe^III^(CN)_6_] is shifted to a much lower extent because the oxidation is not promoted by a mediator. Therefore, a reduction of KNi^II^[Fe^III^(CN)_6_] is possible without an oxidation reaction in the positively going potential scan in the range between +0.3 and +0.5 V.

The appearance of such charge trapping^46,47^ depends critically on the architecture of the film with two stratified layers. As a control, a film was prepared in which Ni-HCF and Fe-HCR were deposited in alternating multilayers, for which the voltammogram is shown in Figure 4d. It exhibits additive signals for Fe-HCR (similar to Figure 4a) and Ni-HCF (similar to Figure 4b). The reduction peak *E*_pc,2_ of Ni-HCF in the mixed material is located at 0.67 V, the difference to the reduction peak of the pure Ni-HCF (*E*_pa,2_ = 0.60 V in Figure 4b) film is small. The peaks at *E*_pc,1_ = 0.24 V and *E*_pa,1_ = 0.31 V belong to the reduction and oxidation of Fe-HCR. The voltammetric behavior promotes the hypothesis that the electron transfer is not influenced by mixing the materials. Both materials behave very similar to the respective pure films in Figure 4a,b.

The dependence of peak currents and peak potentials on the scan rate for the layered sample is demonstrated in Figure S2 and does not show features other than those typically found in metal hexacyanometallates.

### Charge Conductance in the Solid State

Experiments of M^1^-HCM^2^s in the solid state without liquid electrolyte are possible because the network structure contains mixed-valent redox centers, between which charge can be exchanged, as well as partially solvated counter cations that stabilize the different redox states.^46^ However, there is no net flux of K^+^ for charge balancing between the coordination networks and an adjacent ion-conducting phase, as is the case in voltammetry. In order to obtain more information regarding the solid-state electrochemical behavior of our materials, measurements of current–voltage (*I*–*V*) curves were recorded using conductive SFM tips (Figure 5) that allow to address the microscopic region on the sample. By using the widely used abbreviation “*I*–*V* characteristics”, we intend to demark a clear distinction to CV. Here, as “*V* [V]” stands for the bias voltage applied between both contacts (rather than the electrode potential *E* [V] vs a reference electrode in voltammetry). The voltage was applied across the films of the coordination network compounds between a conducting SFM cantilever and the ITO substrate. For each sample, several *I*–*V* curves were recorded (details in Supporting Information 6). All curves are shown in Figure 5 as gray lines. Below we discuss the averaged curves shown in Figure 5 as a thick black line.

The *I*–*V* curve of pure Fe-HCR (Figure 5a) shows a symmetric behavior, which was also reported for the *I*–*V* characteristics of Prussian blue (iron hexacyanoferrate, Fe-HCF),^58^ where the curve was almost flat between −0.5 and +0.5 V and increase linearly between this range and ±3.0 V. For Fe-HCR (Figure 5a), the curve is almost linear between −1 and +1 V followed by a section, where the curve rises exponentially in negative as well as positive directions. The *I*–*V* characteristics change drastically if the materials are arranged in stratified layers (Figure 5b,d).

The sample for Figure 5b was an Fe-HCR thin film, onto which Ni-HCF nanoparticles (TEM images in Figure S8) were drop-casted. Such samples allow recording *I*–*V* curves on the uncovered Fe-HCR films (by placing the C-SFM tip in between nanoparticles) and for the layered system (with the C-SFM tip on an individual Ni-HCF nanoparticle). An overview scan of 15 × 15 μm (Figure S7c) allowed for the locating of single nanoparticles on the Fe-HCR film. Afterward, the scan area was stepwise reduced until the scanned area was smaller than the lateral extension of the nanoparticle. This was evident by the fact that the nanoparticle filled the entire image frame. The *I*–*V* curves in Figure 5b remains flat in the voltage range between ca. −1 and +2 V. This is a larger area than that observed for a pure Fe-HCR film in Figure 5a. The further increase in the current on both sides of the flat region is asymmetrical. At positive bias, the curve increases exponentially, in the negative biased region, a leakage current is detected up to the breakdown voltage at around −4 V (Figure 5b).

The *I*–*V* curves of the mixed material in Figure 5c show strong variations between different locations on the same sample, which is not found in Figure 5a,b. Representative individual curves are shown in Figure S9. Some curves in Figure 5c are similar to those in Figure 5d (curves 1 and 2), and others are similar to an ohmic behavior (curves 3 and 4). This locally different behavior underlines the disordered architecture of the film.

The analysis of the *I*–*V* curves in the solid state (Figure 5b,d) reveals rectifying behavior of the layered material similar to the results obtained by CV (Figure 4c). This phenomenon is not observed if the same elements are processed into a mixed layer. The air-dried samples contain structural water.^46,59^ It is known that the mobility of K^+^ counterions and thus the conductivity depend on the presence of structural water in M^1^-HCM^2^ materials.^46^ In our air-dried sample, this condition is met, and the inner Fe-HCR film, which is in direct contact to the back electrode, is easily reduced if a sufficiently negative potential is applied. The outer Ni-HCF material is in contact with either the electrolyte solution or the tip of the cantilever. In both experiments, the Ni-HCF layer can be reduced only once the reduction of Fe-HCR has commenced. *I*–*V* curve in Figure 5b,d confirms that Ni-HCF does not directly exchange electrons with the back electrode and that the electronic contact between the inner Fe-HCR layer and the outer layer Ni-HCR nanoparticles is established as a result of the preparation method.

The role of film preparation is also evident from the comparison of the *I*–*V* curves for Fe-HCR in Figure 5a and the layered sample Fe-HCR|Ni-HCF (Figure 5d), which showed much higher currents. This phenomenon is discussed in Supporting Information 6. The comparison of SEM images of the two samples (Figure 2a) shows a pronounced granular structure of the Fe-HCR film, which might cause the current to flow over a limited number of grains when contacted by the C-SFM tip (Figure S11a), whereas the smoother film of Ni-HCF (Figure 2b) may be able to distribute the current from the C-SFM tip to a significantly larger number of Fe-HCF grains and thus to enhance the overall current (Figure S11b). This is also supported by the observation that dispersed Ni-HCF crystals used in Figure 5b do not show amplification of the current, in agreement with the assumptions that they contact only a limited number of Fe-HCR grains of the inner layer.

The system Fe-HCR|Ni-HCF worked best among the tested combinations Fe-HCF|Ni-HCF, Fe-HCR|Cu-HCF, and Ni-HCF|Zn-HCF with our preparation techniques. Some results for those combinations are summarized in Supporting Information 8. They highlight the requirement for structural and processing compatibility between the inner and outer layer when used without polymeric binder materials.

## Conclusions

The electronic behavior and electrochemical properties of layered iron hexacyanoruthenate (Fe-HCR) and nickel hexacyanoferrate (Ni-HCF) samples depend strongly on the nanoscale layer architecture. A “mixed layer”—obtained by alternatingly performing an electrochemical synthesis by one oxidation–reduction potential cycle in the respective precursor solution—yields films whose surface voltammetry exhibits a superposition of the signals found in the voltammograms of pure Fe-HCR or pure Ni-HCF films. Such mixed layers do not show a rectifying behavior in *I*–*V* curves recorded between the back Au electrode and a C-SFM tip.

However, rectifying behavior is observed if stratified layers of pure electrodeposited granular Fe-HCR are overcoated by pure Ni-HCF formed in a LbL approach and yield a flaky morphology. In previous literature, metal HCF systems with rectifying behavior have been obtained by connecting and at the same time separating the different metal hexacyanometallate layers by a polymer layer.^46^ In this communication, a direct connection between the two materials was achieved by first electrochemically depositing Fe-HCR and overcoating this by LbL deposition of Ni-HCF. Alternatively, the second layer can be applied as presynthesized Ni-HCF nanoparticles. The latter system allowed us to compare *I*–*V* curves of the layered system Fe-HCR|Ni-HCF with those from Fe-HCR by placing the C-SFM tip either on a Ni-HCF nanoparticle or directly on the Fe-HCR layer. The LbL buildup is a suitable method to form extended stratified layers because there is little impact on the previously deposited layer. This distinguishes this approach from electrodeposition, in which the redox state of the first film is periodically changed and may promote ion-exchanges processes at the interface between the two M^1^-HCM^2^ materials. The electrochromic and electrocatalytic properties of Fe-HCR may thus be modulated not only by electronic communication with the supporting electrode but also may be integrated into a more sophisticated scheme for manipulating and reading the charging state of the material.

## Supporting Information Available

Experimental details of electrodeposition and CV of Fe-HCR, Ni-HCF, and mixed films of both materials; XPS; XRD measurements; SFM measurements; TEM measurements of Ni-HCF nanoparticles; experimental details for recording *I*–*V* curves outside of electrolytes; polarization modulation infrared reflection absorption spectroelectrochemistry; and report on attempts with other layered material combinations (PDF)

## References

[ref1] Thomas S. R.; Pattanasattayavong P.; Anthopoulos T. D. Solution-Processable Metal Oxide Semiconductors for Thin-Film Transistor Applications. Chem. Soc. Rev. 2013, 42, 6910–6923. 10.1039/c3cs35402d. 23770615

[ref2] Stavila V.; Talin A. A.; Allendorf M. D. MOF-Based Electronic and Opto-Electronic Devices. Chem. Soc. Rev. 2014, 43, 5994–6010. 10.1039/C4CS00096J. 24802763

[ref3] Janiak C.; Vieth J. K. MOFs, MILs and More: Concepts, Properties and Applications for Porous Coordination Networks (PCNs). New J. Chem. 2010, 34, 2366–2388. 10.1039/c0nj00275e.

[ref4] Sun L.; Campbell M. G.; Dincă M. Electrically Conductive Porous Metal-Organic Frameworks. Angew. Chem., Int. Ed. 2016, 55, 3566–3579. 10.1002/anie.201506219. 26749063

[ref5] Husmann S.; Zarbin A. J. G. Multifunctional Carbon Nanotubes/ruthenium Purple Thin Films: Preparation, Characterization and Study of Application as Sensors and Electrochromic Materials. Dalton Trans. 2015, 44, 5985–5995. 10.1039/C4DT02784A. 25407673

[ref6] Song X.; Song S.; Wang D.; Zhang H. Prussian Blue Analogs and Their Derived Nanomaterials for Electrochemical Energy Storage and Electrocatalysis. Small Methods 2021, 5, 2001000 10.1002/smtd.202001000. 34927855

[ref7] Shekhah O.; Liu J.; Fischer R. A.; Wöll C. MOF Thin Films: Existing and Future Applications. Chem. Soc. Rev. 2011, 40, 1081–1106. 10.1039/c0cs00147c. 21225034

[ref8] Heinke L.; Wöll C. Surface-Mounted Metal-Organic Frameworks: Crystalline and Porous Molecular Assemblies for Fundamental Insights and Advanced Applications. Adv. Mater. 2019, 31, e1806324 10.1002/adma.201806324. 30701602

[ref9] Ulusoy Ghobadi T. G.; Ozbay E.; Karadas F. How to Build Prussian Blue Based Water Oxidation Catalytic Assemblies: Common Trends and Strategies. Chem. - Eur. J. 2021, 27, 3638–3649. 10.1002/chem.202004091. 33197292

[ref10] Li W.-J.; Han C.; Cheng G.; Chou S.-L.; Liu H.-K.; Dou S.-X. Chemical Properties, Structural Properties, and Energy Storage Applications of Prussian Blue Analogues. Small 2019, 15, 1900470 10.1002/smll.201900470. 30977287

[ref11] Zakaria M. B.; Chikyow T. Recent Advances in Prussian Blue and Prussian Blue Analogues: Synthesis and Thermal Treatments. Coord. Chem. Rev. 2017, 352, 328–345. 10.1016/j.ccr.2017.09.014.

[ref12] Chen C. F.; Wang C. M. Ruthenium Purple-Containing Zeolite Modified Electrodes and Their Application for the Detection of Glucose. J. Electroanal. Chem. 1999, 466, 82–89. 10.1016/S0022-0728(99)00129-1.

[ref13] Jain V.; Sahoo R.; Jinschek J. R.; Montazami R.; Yochum H. M.; Beyer F. L.; Kumar A.; Heflin J. R. High Contrast Solid State Electrochromic Devices Based on Ruthenium Purple Nanocomposites Fabricated by Layer-by-Layer Assembly. Chem. Commun. 2008, 3663–3665. 10.1039/b803915a. 18665292

[ref14] Wessells C. D.; Peddada S. V.; Huggins R. A.; Cui Y. Nickel Hexacyanoferrate Nanoparticle Electrodes for Aqueous Sodium and Potassium Ion Batteries. Nano Lett. 2011, 11, 5421–5425. 10.1021/nl203193q. 22043814

[ref15] Cano A.; Rodríguez-Hernández J.; Reguera L.; Rodríguez-Castellón E.; Reguera E. On the Scope of XPS as Sensor in Coordination Chemistry of Transition Metal Hexacyanometallates. Eur. J. Inorg. Chem. 2019, 2019, 1724–1732. 10.1002/ejic.201801556.

[ref16] Buser H. J.; Schwarzenbach D.; Petter W.; Ludi A. The crystal structure of Prussian Blue: Fe4[Fe(CN)6]3.xH2O. Inorg. Chem. 1977, 16, 2704–2710. 10.1021/ic50177a008.

[ref17] Rudola A.; Du K.; Balaya P. Monoclinic Sodium Iron Hexacyanoferrate Cathode and Non-Flammable Glyme-Based Electrolyte for Inexpensive Sodium-Ion Batteries. J. Electrochem. Soc. 2017, 164, A1098 10.1149/2.0701706jes.

[ref18] Karyakin A. A. Prussian Blue and Its Analogues: Electrochemistry and Analytical Applications. Electroanalysis 2001, 13, 813–819. 10.1002/1521-4109(200106)13:10<813::AID-ELAN813>3.0.CO;2-Z.

[ref19] Itaya K.; Uchida I.; Neff V. D. Electrochemistry of Polynuclear Transition Metal Cyanides: Prussian Blue and Its Analogues. Acc. Chem. Res. 1986, 19, 162–168. 10.1021/ar00126a001.

[ref20] Coon D. R.; Amos L. J.; Bocarsly A. B.; Fitzgerald Bocarsly P. A. Analytical Applications of Cooperative Interactions Associated with Charge Transfer in Cyanometalate Electrodes: Analysis of Sodium and Potassium in Human Whole Blood. Anal. Chem. 1998, 70, 3137–3145. 10.1021/ac970975a. 11013718

[ref21] Lenarczuk T.; Głąb S.; Koncki R. Application of Prussian Blue-Based Optical Sensor in Pharmaceutical Analysis. J. Pharm. Biomed. Anal. 2001, 26, 163–169. 10.1016/S0731-7085(01)00398-3. 11451654

[ref22] Chaudhary A.; Ghosh T.; Pathak D. K.; Kandpal S.; Tanwar M.; Rani C.; Kumar R. Prussian blue-based inorganic flexible electrochromism glucose sensor. IET Nanodielectr. 2021, 4, 165–170. 10.1049/nde2.12011.

[ref23] Huang B.; Shao Y.; Liu Y.; Lu Z.; Lu X.; Liao S. Improving Potassium-Ion Batteries by Optimizing the Composition of Prussian Blue Cathode. ACS Appl. Energy Mater. 2019, 2, 6528–6535. 10.1021/acsaem.9b01097.

[ref24] Lu Y.; Wang L.; Cheng J.; Goodenough J. B. Prussian Blue: A New Framework of Electrode Materials for Sodium Batteries. Chem. Commun. 2012, 48, 6544–6546. 10.1039/c2cc31777j. 22622269

[ref25] Hurlbutt K.; Wheeler S.; Capone I.; Pasta M. Prussian Blue Analogs as Battery Materials. Joule 2018, 2, 1950–1960. 10.1016/j.joule.2018.07.017.

[ref26] Kaye S. S.; Long J. R. Hydrogen Storage in the Dehydrated Prussian Blue Analogues M3[Co(CN)6]2 (M = Mn, Fe, Co, Ni, Cu, Zn). J. Am. Chem. Soc. 2005, 127, 6506–6507. 10.1021/ja051168t. 15869251

[ref27] Natesakhawat S.; Culp J. T.; Matranga C.; Bockrath B. Adsorption Properties of Hydrogen and Carbon Dioxide in Prussian Blue Analogues M3[Co(CN)6]2, M = Co, Zn. J. Phys. Chem. C 2007, 111, 1055–1060. 10.1021/jp065845x.

[ref28] Kaye S. S.; Long J. R. The Role of Vacancies in the Hydrogen Storage Properties of Prussian Blue Analogues. Catal. Today 2007, 120, 311–316. 10.1016/j.cattod.2006.09.018.

[ref29] Itaya K.; Shoji N.; Uchida I. Catalysis of the Reduction of Molecular Oxygen to Water at Prussian Blue Modified Electrodes. J. Am. Chem. Soc. 1984, 106, 3423–3429. 10.1021/ja00324a007.

[ref30] Zhao C.; Liu B.; Li X.; Zhu K.; Hu R.; Ao Z.; Wang J. A Co-Fe Prussian Blue Analogue for Efficient Fenton-Like Catalysis: The Effect of High-Spin Cobalt. Chem. Commun. 2019, 55, 7151–7154. 10.1039/C9CC01872G. 31119224

[ref31] Yu Z.-Y.; Duan Y.; Liu J.-D.; Chen Y.; Liu X.-K.; Liu W.; Ma T.; Li Y.; Zheng X.-S.; Yao T.; Gao M.-R.; Zhu J.-F.; Ye B.-J.; Yu S.-H. Unconventional CN Vacancies Suppress Iron-Leaching in Prussian Blue Analogue Pre-Catalyst for Boosted Oxygen Evolution Catalysis. Nat. Commun. 2019, 10, 2799 10.1038/s41467-019-10698-9. 31243269 PMC6595008

[ref32] Sitnikova N. A.; Komkova M. A.; Khomyakova I. V.; Karyakina E. E.; Karyakin A. A. Transition Metal Hexacyanoferrates in Electrocatalysis of H2O2 Reduction: An Exclusive Property of Prussian Blue. Anal. Chem. 2014, 86, 4131–4134. 10.1021/ac500595v. 24735447

[ref33] Kelly M. T.; Arbuckle-Keil G. A.; Johnson L. A.; Su E. Y.; Amos L. J.; Chun J. K. M.; Bocarsly A. B. Nickel Ferrocyanide Modified Electrodes as Active Cation-Exchange Matrices: Real Time XRD Evaluation of Overlayer Structure and Electrochemical Behavior. J. Electroanal. Chem. 2001, 500, 311–321. 10.1016/S0022-0728(00)00487-3.

[ref34] Thammawong C.; Opaprakasit P.; Tangboriboonrat P.; Sreearunothai P. Prussian Blue-Coated Magnetic Nanoparticles for Removal of Cesium from Contaminated Environment. J. Nanopart. Res. 2013, 15, 1689 10.1007/s11051-013-1689-z.

[ref35] Feng S.; Li X.; Ma F.; Liu R.; Fu G.; Xing S.; Yue X. Prussian Blue Functionalized Microcapsules for Effective Removal of Cesium in a Water Environment. RSC Adv. 2016, 6, 34399–34410. 10.1039/C6RA01450J.

[ref36] Rìos C.; Monroy-Noyola A. D-Penicillamine and Prussian Blue as Antidotes Against Thallium Intoxication in Rats. Toxicology 1992, 74, 69–76. 10.1016/0300-483X(92)90044-F. 1514189

[ref37] Shokouhimehr M.; Soehnlen E. S.; Hao J.; Griswold M.; Flask C.; Fan X.; Basilion J. P.; Basu S.; Huang S. D. Dual Purpose Prussian Blue Nanoparticles for Cellular Imaging and Drug Delivery: A New Generation of T1-Weighted MRI Contrast and Small Molecule Delivery Agents. J. Mater. Chem. 2010, 20, 5251–5259. 10.1039/b923184f.

[ref38] de Tacconi N. R.; Rajeshwar K.; Lezna R. O. Metal Hexacyanoferrates: Electrosynthesis, in Situ Characterization, and Applications. Chem. Mater. 2003, 15, 3046–3062. 10.1021/cm0341540.

[ref39] Widmann A.; Kahlert H.; Petrovic-Prelevic I.; Wulff H.; Yakhmi J. V.; Bagkar N.; Scholz F. Structure, Insertion Electrochemistry, and Magnetic Properties of a New Type of Substitutional Solid Solutions of Copper, Nickel, and Iron Hexacyanoferrates/Hexacyanocobaltates. Inorg. Chem. 2002, 41, 5706–5715. 10.1021/ic0201654. 12401075

[ref40] Chen S.-M. Characterization and Electrocatalytic Properties of Cobalt Hexacyanoferrate Films. Electrochim. Acta 1998, 43, 3359–3369. 10.1016/S0013-4686(98)00074-7.

[ref41] Hosseini P.; Wolkersdörfer K.; Wark M.; Redel E.; Baumgart H.; Wittstock G. Morphology and Conductivity of Copper Hexacyanoferrate Films. J. Phys. Chem. C 2020, 124, 16849–16859. 10.1021/acs.jpcc.0c06114.

[ref42] Harms L.; Roth N.; Wittstock G. A New Programmable Dipping Robot. Electrochem. Sci. Adv. 2023, 3, e2100177 10.1002/elsa.202100177.

[ref43] Li C. H.; Nanba Y.; Asakura D.; Okubo M.; Talham D. R. Li-Ion and Na-Ion Insertion into Size-Controlled Nickel Hexacyanoferrate Nanoparticles. RSC Adv. 2014, 4, 24955–24961. 10.1039/c4ra03296a.

[ref44] Grandjean F.; Samain L.; Long G. J. Characterization and Utilization of Prussian Blue and Its Pigments. Dalton Trans. 2016, 45, 18018–18044. 10.1039/C6DT03351B. 27801448

[ref45] Long X.; Chen R.; Yang S.; Wang J.; Huang T.; Lei Q.; Tan J. Preparation, Characterization and Application in Cobalt Ion Adsorption Using Nanoparticle Films of Hybrid Copper-nickel Hexacyanoferrate. RSC Adv. 2019, 9, 7485–7494. 10.1039/C9RA00596J. 35519994 PMC9061196

[ref46] Miecznikowski K.; Chojak M.; Steplowska W.; Malik M.; Kulesza P. Microelectrochemical Electronic Effects in Two-Layer Structures of Distinct Prussian Blue Type Metal Hexacyanoferrates. J. Solid State Electrochem. 2004, 8, 868–875. 10.1007/s10008-004-0555-4.

[ref47] Ono K.; Ishizaki M.; Soma S.; Kanaizuka K.; Togashi T.; Kurihara M. A Low-Temperature Sintered Heterostructure Solid Film of Coordination Polymer Nanoparticles: An Electron-Rectifier Function Based on Partially Oxidised/reduced Conductor Phases of Prussian Blue. RSC Adv. 2015, 5, 96297–96304. 10.1039/C5RA18678A.

[ref48] Karpova E. V.; Karyakina E. E.; Karyakin A. A. Iron-nickel hexacyanoferrate bilayer as an advanced electrocatalyst for H2O2reduction. RSC Adv. 2016, 6, 103328–103331. 10.1039/C6RA24128J.

[ref49] Abruña H. D.; Denisevich P.; Umana M.; Meyer T. J.; Murray R. W. Rectifying Interfaces Using Two-Layer Films of Electrochemically Polymerized Vinylpyridine and Vinylbipyridine Complexes of Ruthenium and Iron on Electrodes. J. Am. Chem. Soc. 1981, 103, 1–5. 10.1021/ja00391a001.

[ref50] Abe T.; Toda G.; Tajiri A.; Kaneko M. Electrochemistry of Ferric Ruthenocyanide (Ruthenium Purple), and Its Electrocatalysis for Proton Reduction. J. Electroanal. Chem. 2001, 510, 35–42. 10.1016/S0022-0728(01)00539-3.

[ref51] De Benedetto G. E.; Guascito M. R.; Ciriello R.; Cataldi T. R. I. Analysis by X-Ray Photoelectron Spectroscopy of Ruthenium Stabilised Polynuclear Hexacyanometallate Film Electrodes. Anal. Chim. Acta 2000, 410, 143–152. 10.1016/S0003-2670(00)00724-8.

[ref52] Weidinger D.; Brown D. J.; Owrutsky J. C. Transient Absorption Studies of Vibrational Relaxation and Photophysics of Prussian Blue and Ruthenium Purple Nanoparticles. J. Chem. Phys. 2011, 134, 124510 10.1063/1.3564918. 21456679

[ref53] Gupta R. P.; Sen S. K. Calculation of multiplet structure of corep-vacancy levels. II. Phys. Rev. B: Solid State 1975, 12, 15–19. 10.1103/PhysRevB.12.15.

[ref54] Cataldi T. R. I.; Salvi A. M.; Centonze D.; Sabbatini L. Voltammetric and XPS Investigations of Polynuclear Ruthenium-Containing Cyanometallate Film Electrodes. J. Electroanal. Chem. 1996, 406, 91–99. 10.1016/0022-0728(95)04426-4.

[ref55] Malik M. A.; Kulesza P. J.; Wlodarczyk R.; Wittstock G.; Szargan R.; Bala H.; Galus Z. Formation of Ultra-Thin Prussian Blue Layer on Carbon Steel that Promotes Adherence of Hybrid Polypyrrole Based Protective Coating. J. Solid State Electrochem. 2005, 9, 403–411. 10.1007/s10008-005-0654-x.

[ref56] Zamponi S.; Berrettoni M.; Kulesza P. J.; Miecznikowski K.; Malik M. A.; Makowski O.; Marassi R. Influence of Experimental Conditions on Electrochemical Behavior of Prussian Blue Type Nickel Hexacyanoferrate Film. Electrochim. Acta 2003, 48, 4261–4269. 10.1016/j.electacta.2003.08.001.

[ref57] Chen W.; Tang J.; Xia X.-H. Composition and Shape Control in the Construction of Functional Nickel Hexacyanoferrate Nanointerfaces. J. Phys. Chem. C 2009, 113, 21577–21581. 10.1021/jp908112u.

[ref58] Xidis A.; Neff V. D. On the Electronic Conduction in Dry Thin Films of Prussian Blue, Prussian Yellow, and Everitt’s Salt. J. Electrochem. Soc. 1991, 138, 3637–3642. 10.1149/1.2085472.

[ref59] Kulesza P. J. Solid-state electrochemistry of iron hexacyanoferrate (Prussian Blue type) powders. J. Electroanal. Chem. Interfacial Electrochem. 1990, 289, 103–116. 10.1016/0022-0728(90)87209-3.

