# Supplementary material for: Unidirectional Current in Layered Metal Hexacyanometallate Thin Films: Implication for Alternative Wet-Processed Electronic Materials
Source: ACS Omega. 2023 Nov 8;8(46):44139–47. doi: 10.1021/acsomega.3c06447 (PMC10666236; doi:10.1021/acsomega.3c06447)
Supplement: Supplementary file 1 — ao3c06447_si_001.pdf [file ao3c06447_si_001.pdf]

Unidirectional Current in Layered Metal Hexacyanometallate Thin Films: Implication for Alternative Wet-processed Electronic Materials

--Supporting Information --

Lena Gerhards, Gunther Wittstock*

Carl von Ossietzky University of Oldenburg, School of Mathematics and Science, Institute of Chemistry, 26111 Oldenburg, Germany

CORRESPONDING AUTHOR EMAIL ADRESS: wittstock@uol.de

Content

SI-1 Electrochemical Deposition and Cyclic Voltammetry of Fe-HCR, Ni-HCF and the Mixed Material S-3

SI-1.1 Further Preparation Details S-3

SI-1.2 Characterization of Metalhexacyanometallate Films by Cyclic Voltammetry in Supporting Electrolyte S-5

SI-2 XPS Measurements S-7

SI-2.1 Fitting of Fe 2p Signals of Stratified Fe-HCR|Ni-HCF Layers, Mixed Material of Fe-HCR and Ni-HCF, and Fe-HCR S-7

SI-2.2 K 2p Signals of Stratified Fe-HCR|Ni-HCF Layers, Mixed Material of Fe-HCR and Ni-HCF, and Fe-HCR S-8

SI-2.3 N 1s Signals of Stratified Fe-HCR|Ni-HCF Layers, Mixed Material of Fe-HCR and Ni-HCF, and Fe-HCR S-9

SI-2.4 Reference Spectra of $K_4[Ru(CN)_6]$ S-11

SI-2.5 Reference Spectra of Ni-HCF Nanoparticles S-13

SI-3 XRD Measurements S-15

SI-4 SFM Measurements S-16

SI-5 TEM Measurements of Ni-HCF Nanoparticles S-18

SI-6 Recording of *I-V* Curves S-20

SI-6.1 Experimental Procedure S-20

SI-6.2 Details of *I-V* Curves S-20

SI-6.3 Comparison of Currents in I-V curves on Fe-HCR and Fe-HCR|Ni-HCF S-22

SI-7 In-situ Polarization Modulation Infrared Reflection Absorption Spectroscopy S-24

SI-7.1 Experimental Details S-24

SI-7.2 Fe-HCR S-24

SI-7.3 Ni-HCF S-26

SI-7.4 Fe-HCR/Ni-HCF Layered Material S-28

SI-8 Attempts on Other Layered Systems S-30

SI-8.1 Fe-HCR | Cu-HCF S-30

SI-8.2 Systems with Zn-HCF S-31

SI-8.3 Fe-HCF|Ni-HCF S-33

References S-34

SI-1 Electrochemical Deposition and Cyclic Voltammetry of Fe-HCR, Ni-HCF and the Mixed Material

SI-1.1 Further Preparation Details

Fe-HCR was electrochemically deposited during 15 potential cycles in the potential window -0.2 to 0.6 V (ag|AgCl|3 mol L^{-1} KCl) with a scan rate v = 40 mV s^{-1} in an aqueous solution of 1 mmol L^{-1} $K_4[Ru(CN)_6]$ + 1 mmol L^{-1} $FeCl_3$ + 70 mmol L^{-1} KCl (Figure S1a). Ni-HCF was electrochemically deposited during 15 potential cycles in the potential window 0.0 to 0.75 V with a scan rate of v = 40 mV s^{-1} in an aqueous solution of 1 mmol L^{-1} $NiCl_2$ + 0.5 mmol L^{-1} $K_3[Fe(CN)_6]$ + 500 mmol L^{-1} KCl (Figure S1b). An increase in peak current in the cyclic voltammograms of Figure S1a-c indicates an incremental growth of the films on the electrode surface in each potential cycle.

The mixed material of Fe-HCR and Ni-HCF was obtained by executing one potential cycle in the range -0.2 to 0.6 V with a scan rate v = 40 mV s^{-1} in an aqueous solution of 1 mmol L^{-1} $K_4[Ru(CN)_6]$ + 1 mmol L^{-1} $FeCl_3$ + 70 mmol L^{-1} KCl. The substrate was then removed from the solution, rinsed and transferred to aqueous 1 mmol L^{-1} $NiCl_2$ + 0.5 mmol L^{-1} $K_3[Fe(CN)_6]$ + 500 mmol L^{-1} KCl, in which one potential cycle was executed in the range 0.0 to 0.75 V with a scan rate of v = 40 mV s^{-1}. After emersing and rinsing the sample, the sequence was repeated 14 times. Figure S1c shows the deposition cycles and the incremental growth of the deposited materials. Curves 1 (in blue) are the deposition of Fe-HCR and the curves 2 (in black) are the deposition cycles for Ni-HCF.

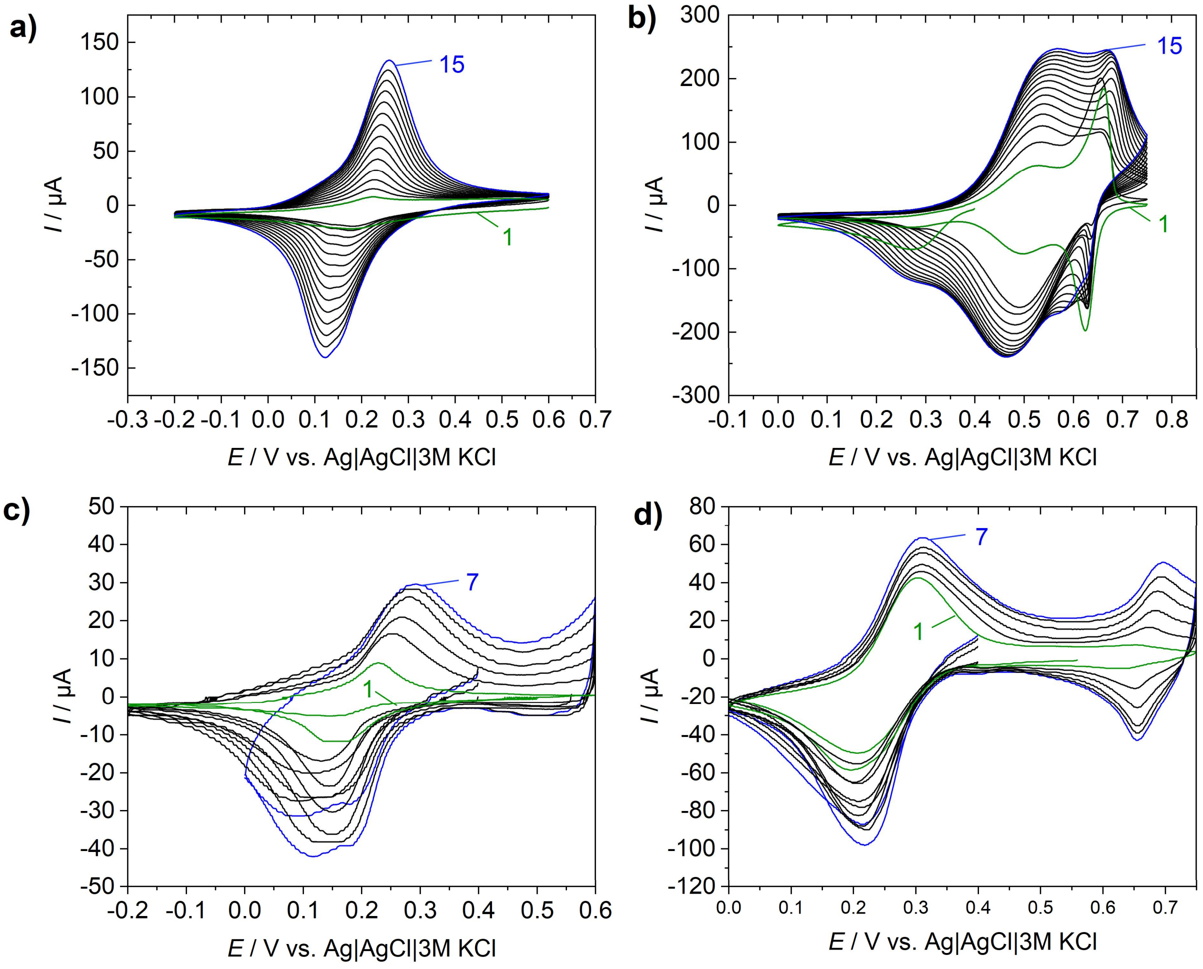

Figure S1. Electrochemical deposition of a) Fe-HCR; b) Ni-HCF; c) and d) mixed material, (c) selected cycles of Fe-HCR deposition and (d) selected cycles of Ni-HCF deposition on ITO. Scan rate is 40 mV s^{-1}. The numbers indicate the potential cycle. The first and last potential cycles are indicated in color.

The formal potential $E^{\circ\prime}$ was calculated as the mean of anodic and cathodic peak potentials:

$$E^{\circ\prime} = \frac{E_{pa}+E_{pc}}{2} \tag{S1}$$

For Ni-HCF, the marked peak pair in Figure 4b was selected. According to literature,[1,2] it corresponds to the redox process:

$$KNi^{II}[Fe^{III}(CN)_6] + e + K^+ \rightarrow K_2Ni^{II}[Fe^{II}(CN)_6] \tag{S2}$$

The exact peak positions are listed in Tables S1 to S4

Table S1. Anodic and cathodic peak potentials of Fe-HCR (Figure 4a)

Peak	E / V
E_{pa}	0.31
E_{pc}	0.21

Table S2. Anodic and cathodic peak potentials of Ni-HCF (marked in Figure 4b)

Peak	E / V
E_{pa}	0.72
E_{pc}	0.63

Table S3. Anodic and cathodic peak potentials of the layered material with Fe-HCR as inner layer and Ni-HCF as outer layer (Figure 4c)

Peak	E / V
$E_{pa,1}$	0.36
$E_{pc,1}$	0.19
$E_{pa,2}$	0.60
$E_{pc,2}$	0.39

Table S4. Anodic and cathodic peak potentials of the mixed material of Fe-HCR and Ni-HCF (Figure 4d)

Peak	E / V
$E_{pa,1}$	0.31
$E_{pc,1}$	0.24
$E_{pa,2}$	0.71
$E_{pc,2}$	0.67

SI-1.2 Characterization of Metalhexacyanometallate Films by Cyclic Voltammetry in Supporting Electrolyte

The dependence of the peak current on scan rate v was investigated for Fe-HCR|Ni-HCF layered films (Figure S2). The plot of the peak current vs. the square root of the scan rate is almost linear (Figure S2) indicated the control by a diffusion process (diffusion of charge balancing K^+). The plot of peak potentials vs. scan rate shows a trend for increasing the peak separation with scan rate without changing the overall qualitative picture.

Please note, that the data on Figure S2 were acquired on a different sample and with a different capillary in an in-house built droplet cell than Figure 4c of the main manuscript. Therefore, the area of the sample wetted by the droplet is different and the voltammetric currents in Figure S2 vs. Figure 4c are not comparable. We also noted that the addressed amount of material may differ even for different landing sites on the same sample and with the same capillary. The different amount of material addressed also causes different resistivity effects in the sample material that causes slight shifts in peak potentials.

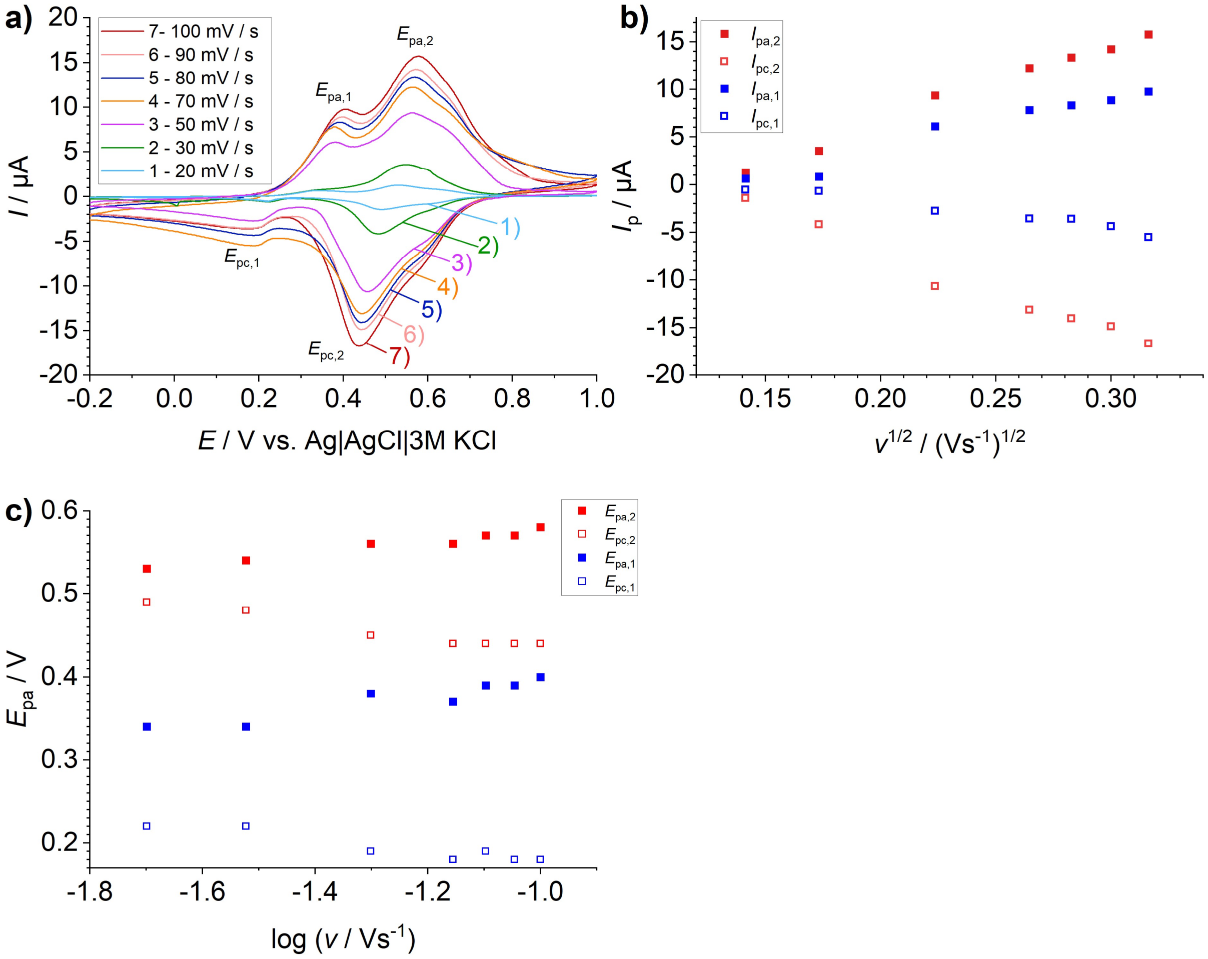

Figure S2. a) Cyclic voltammetry of Fe-HCR|Ni-HCF in 1 mol L^{-1} KCl at different scan rates 1) 20 mV s^{-1}, 2) 30 mV s^{-1}, 3) 50 mV s^{-1}, 4) 70 mV s^{-1}, 5 80 mV s^{-1}, 6) 90 mV s^{-1}, 7) 100 mV s^{-1}; b) Plot of the peak current vs. square root of the scan rate; c) plot of peak potentials vs. log(*v*).

SI-2 XPS Measurements

SI-2.1 Fitting of Fe 2p Signals of Stratified Fe-HCR|Ni-HCF Layers, Mixed Material of Fe-HCR and Ni-HCF, and Fe-HCR

The Fe $2p_{3/2}$ signal can be fitted with a multiplet as theoretically calculated by Gupta and Sen[3] using Hartree-Fock approximation for free ions with the same number of unpaired 3d electrons as in considered emitter atom. This is isolated Fe^{2+} for Fe^{2+} high-spin and isolated Fe^{3+} for Fe^{3+} high-spin and Cr^{5+} for Fe^{3+} low-spin.

The Fe $2p_{3/2}$ spectrum of Fe-HCR (Figure 3a) indicates the presence of high-spin Fe^{2+} with three components at E_B = {709.5, 710.8, 711.9} in an intensity ratio of 78 : 100 : 40, a surface peak and satellite peak (assignment in Table S7).[4] This assignment is in agreement with previous findings that the N-coordinated transition metal (i.e., Fe in Fe-HCR, Figure 3a) is preferentially in the high-spin configuration,[5,6] while the C-coordinated metal ion is in the low-spin configuration as found for Fe^{2+} in Ni-HCF (Figure 3c, e). Since all six Fe 3d electrons are paired, a single Fe $2p_{3/2}$ component at E_B = 708.5 eV is recorded without multiplet splitting.[7] The expected valence state of iron is Fe^{2+} in the mixed material because the electrochemical deposition cycle was stopped after reduction (-0.2 V vs Ag|AgCl|3 mol L^{-1} KCl). Note, that the expected Fe^{2+} high-spin from Fe-HCR in Figure 3c is only visible as a small signal. Several reasons might be responsible for this. The current in the electrodeposition experiments indicates that the Ni-HCF films grow more rapidly than the Fe-HCR film. This could lead to rapid coverage of the material so that only a few high-spin Fe^{2+} ions are located within the information depth of XPS. Furthermore, there might be a change from high-spin to low-spin configuration in the mixed materials in which the coordination environment is less defined than in a pure material.

The signal for Fe^{2+} low-spin of the mixed material is also found for a pure Ni-HCF sample (Figure S5). In addition, the Fe $2p_{3/2}$ spectrum in Figure S5 contains a multiplet indicative of additional Fe^{3+} low-spin in the Ni-HCF sample.

In the layered material there are two different iron centers, in Ni-HCF and in the covered film of Fe-HCR. The spectrum in (Figure 3e) shows only the signal for Fe in Ni-HCF. This can be concluded from the complete absence of the signal for Ru $3d_{5/2}$ at E_B = 281.0 in Figure 3f. This conclusion is in agreement with the film thickness of the Ni-HCF film (ca. 110 nm), which is greater than the information depth of XPS. The peak at E_B = 710.2 eV and associated multiplet components in Figure 3e likely originate from Fe^{3+} species in the material, which may originate from a partial oxidation during preparation in a lengthy layer-by-layer procedure. Due to the low intensity of the high E_B components, a safe distinction between Fe^{3+} and high-spin Fe^{2+} cannot be made with certainty for this overlaid signal. The Fe $2p_{3/2}$ signal in Figure 3e contains a single component from low-spin Fe^{2+} (also found in Figure 3c).

SI-2.2 K 2p Signals of Stratified Fe-HCR|Ni-HCF Layers, Mixed Material of Fe-HCR and Ni-HCF, and Fe-HCR

Figure 3b, 3d and 3f show doublets for K $2p_{3/2}$ and K $2p_{1/2}$ photoemissions (Tables S5-S7). Two doublets can very clearly be seen in Figure 3d (mixed material) with K $2p_{3/2}$ binding energies of 293.0 eV and 293.9 eV. In Figure 3b and 3f only one doublet can be identified clearly although the slightly broader K $2p_{3/2}$ peak in Figure 3b (full width at half maximum (FWHM) of 1.49 eV compared to 1.05 eV in Figure 3f) may suggest the existence of two different species, whose photoemission peaks could not be resolved spectroscopically. The value for E_B is 293.8 eV in Figure 3f and thus equal within the uncertainty range of the method and also equal to the high binding energy component in Figure 3d. Typical values reported in literature are approximately 293.9 eV.[8,9] The second K $2p_{3/2}$ signal likely originates from Fe-HCR. The K $2p_{3/2}$ binding energy of a $K_4[Ru^{II}(CN)_6] \times x\, H_2O$ is found at 293.00 eV (Figure S4a). A further reason for slightly different binding energies of the charge balancing K^+ could be different hydration states of the material, especially in the vicinity of defects. Those defects might be much more common in the mixed material.

SI-2.3 N 1s Signals of Stratified Fe-HCR|Ni-HCF Layers, Mixed Material of Fe-HCR and Ni-HCF, and Fe-HCR

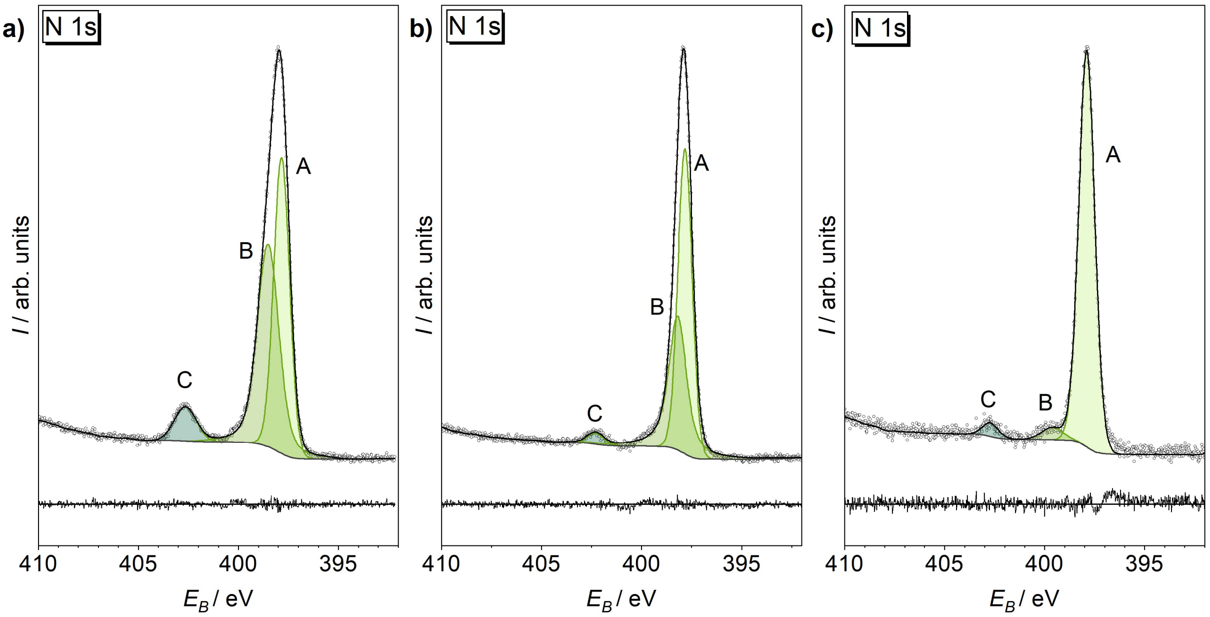

Figure S3. High resolution N 1s XP spectra of a) layered material Fe-HCR|Ni-HCF, b) mixed material of Fe-HCR and Ni-HCF, c) Fe-HCR.

The complete assignments of the XP peaks in Figure S3 and Figure 3 of the main text are summarized in Tables S5-S7.

The component labelled 'A' Is the most intensive signal. The binding energies are equal for the three samples within the accuracy of the method [397.85 eV (Figure S3a), 397.82 eV (Figure S3b) and 397.85 eV (Figure S3c)]. It is likely due to different coordination environment of the CN^- ligand, e.g., Ru^{II}-C<u>N</u>-Fe^{II}, Fe^{II}-C<u>N</u>-Ni^{II}. The component labelled 'B' occurs at 398.46 eV (Figure S3a) and 398.14 eV (Figure S3b) in the Ni-containing materials and, thus, is likely caused by N-atoms in Fe^{III}-C<u>N</u>-Ni^{II} coordination in agreement with the Fe 2p spectra in Figure 3. Differences in binding energies result from differences in π-back bonding from the transition metal cation to the CN ligand for different metals and different oxidation states.[10] The assignment of component C is not absolutely clear. It could be a shake-up satellite for a π-π* excitation in the cyanide ligand upon photoemission from N,[11] or due to oxidized nitrogen species that may be formed at the surface of samples when in contact with air.[12,13]

Table S5. Fitted peaks of high-resolution XP spectra of layered material

E_B[eV]	Assignment	Reference
Fe $2p_{3/2}$		
708.5	low-spin Fe^{2+}	4,14
710.2	Fe^{3+} species	3,14
711.4	Fe^{3+} species	3,14
712.6	Fe^{3+} species	3,14
Ru 3d		
-	-	
-	-	
C 1s		
284.8	CN, adventitious carbon contaminations	10
285.6	sp^2 carbon-nitrogen bonding	10
288.8	shake-up satellite $\pi \rightarrow \pi^*$	10
K 2p		
293.8	K^+	8
296.6	K^+	8
N 1s		
397.8	Fe^{II}-C<u>N</u>-Ni^{II}	10
398.5	Fe^{III}-C<u>N</u>-Ni^{II}	10
402.6	shake-up satellite $\pi \rightarrow \pi^*$ or <u>N</u>O	8,11,13

Table S6. Fitted peaks of high-resolution XP spectra of mixed material.

E_B[eV]	Assignment	Reference
Fe $2p_{3/2}$		
708.5	low-spin Fe^{2+}	4,14
709.4	high-spin Fe^{2+}	3,4
710.4	high-spin Fe^{2+}	3,4
711.4	high-spin Fe^{2+}	3,4
712.9		
Ru 3d		
281.0	Ru^{2+} ($Ru3d_{5/2}$)	15
285.1	Ru^{2+} ($Ru3d_{3/2}$)	15
C 1s		
284.8	CN, adventitious carbon contaminations	10
286.2	sp^2 carbon-nitrogen bonding	10
289.0	shake-up satellite $\pi \rightarrow \pi^*$	10
K 2p		
293.0	K^+-species $K_4[Ru(CN)_6] \times xH_2O$ (K $2p_{3/2}$)	8,11
295.7	K^+-species $K_4[Ru(CN)_6] \times xH_2O$ (K $2p_{1/2}$)	8,11
293.9	K^+ (K $2p_{3/2}$)	8
296.7	K^+ (K $2p_{1/2}$)	8
N 1s		
397.8	Fe^{II}-C<u>N</u>-Ni^{II}, Ru^{II}-C<u>N</u>-Fe^{II}	10
398.1	Fe^{III}-C<u>N</u>-Ni^{II}	10
402.3	shake-up satellite $\pi \rightarrow \pi^*$ or <u>N</u>O	8,11,13

Table S7. Fitted peaks of high resolution XP spectra of Fe-HCR

E_B[eV]	Assignment	Reference
Fe $2p_{3/2}$		
709.5	high-spin Fe^{2+}	3,4
710.8	high-spin Fe^{2+}	3,4
711.9	high-spin Fe^{2+}	3,4
713.0	Surface peak	3,4
715.0	Satellite	4
Ru 3d		
281.0	Ru^{2+} (Ru3$d_{5/2}$)	15
285.1	Ru^{2+} (Ru3$d_{3/2}$)	15
C 1s		
284.8	CN, adventitious carbon contaminations	10
286.4	sp^2 carbon-nitrogen bonding	10
289.7	shake-up satellite $\pi\rightarrow\pi^*$	10
K 2p		
293.8	K^+ (K $2p_{3/2}$)	8
296.5	K^+ (K $2p_{1/2}$)	8
N 1s		
397.9	Ru^{II}-C$\underline{N}$-Fe^{II}	10
399.6	Ru^{II}-C$\underline{N}$-Fe^{III}	10
402.7	shake-up satellite $\pi\rightarrow\pi^*$ or $\underline{N}$O	8,11,13

SI-2.4 Reference Spectra of $K_4[Ru(CN)_6]$

Reference spectra were taken from powder samples of $K_4[Ru(CN)_6] \times \cdot x H_2O$ (Figure S4). The high resolution N 1s spectrum also shows the component C at 402.4 eV in Figure S4b for surface-oxidized CN groups similar to features in Figure S3c and ref 12. The signals located at 397.8 eV can be associated with N-atoms of cyanide coordinated to Ru.[10] The origin of the very small component B is unclear at the moment.

The Ru $3d_{5/2}$ XP spectrum in Figure S4a can be fitted with a signal at 281.0 eV. This signal can be attributed to Ru^{2+}-species in the material. This value is in good agreement to reference measurements of Cataldi et al.[15], where the Ru $3d_{5/2}$ signal was found at 280.9 eV.

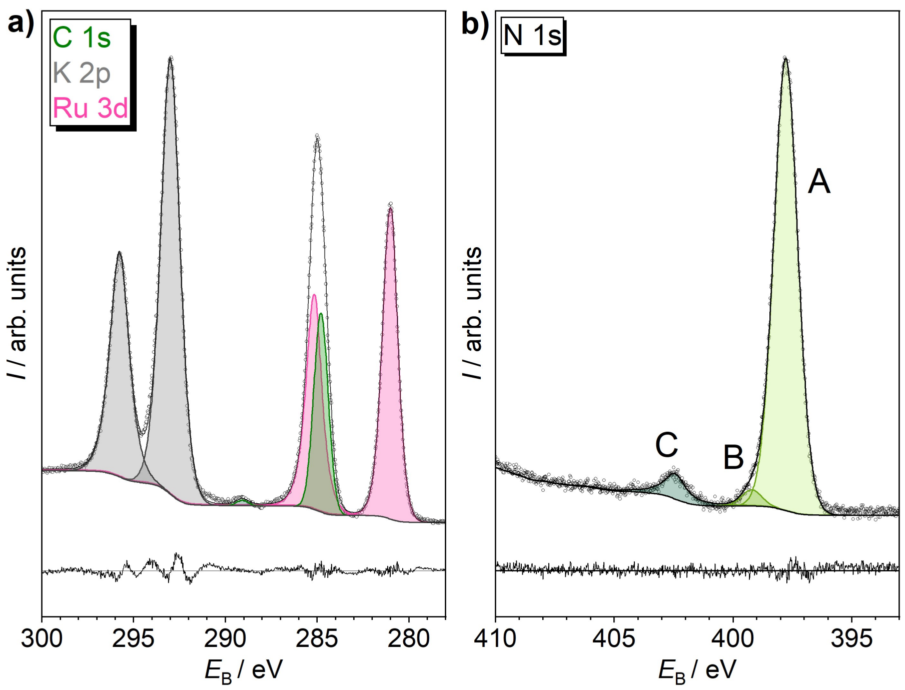

Figure S4. High resolution XP spectra of a) C 1s, K 2p, Ru 3d; and b) N 1s of $K_4[Ru(CN)_6] \times xH_2O$.

Table S8. Fitted peaks of high resolution XP spectra of $K_4[Ru(CN)_6] \times xH_2O$.

E_B[eV]	Assignment	Reference
Ru 3d		
281.0	Ru^{2+} (Ru $3d_{5/2}$)	[15]
285.2	Ru^{2+} (Ru $3d_{3/2}$)	[15]
C 1s		
284.8	CN, adventitious carbon contaminations	[10]
289.1	shake-up $\pi \rightarrow \pi^*$	[10]
K 2p		
293.0	K^+-species $K_4[Ru(CN)_6] \times xH_2O$ (K $2p_{3/2}$)	[8,11]
295.8	K^+-species $K_4[Ru(CN)_6] \times xH_2O$ (K $2p_{1/2}$)	[8,11]
N 1s		
397.8	Ru^{II}-CN	[10]
399.2		[10]
402.4	shake-up satellite $\pi \rightarrow \pi^*$ or <u>N</u>O	[8,11,13]

SI-2.5 Reference Spectra of Ni-HCF Nanoparticles

Reference spectra were taken from Ni-HCF nanoparticles. A drop of the nanoparticle suspension was applied to a gold substrate and allowed to dry under an Ar stream. The sample was then transferred to the ultrahigh vacuum and characterized by XPS. The binding energies of the components are listed in Tables S9.

Of particular interest is the multiplet splitting on the Fe $2p_{3/2}$ spectrum of Ni-HCF nanoparticles in Figure S5. The signal at 708.7 eV can be assigned to low-spin Fe^{2+}. The remaining signal components belong to a multiplet that is formed for photoemission of open shell transition metal ions, here either low-spin Fe^{3+} or high-spin Fe^{2+}.[3,7] A distinction can be made by the analysis of the intensity ratios within the multiplet. The signals at 710.0 eV (100% intensity), 710.6 eV (58% intensity) and 711.5 eV (16% intensity) belong to low-spin Fe^{3+} configuration and produce a good fit.[3] The remaining component at 711.9 eV is a satellite. These XP binding energies for Ni-HCF nanoparticles are in good agreement to data from thick Ni-HCF layers in literature.[7]

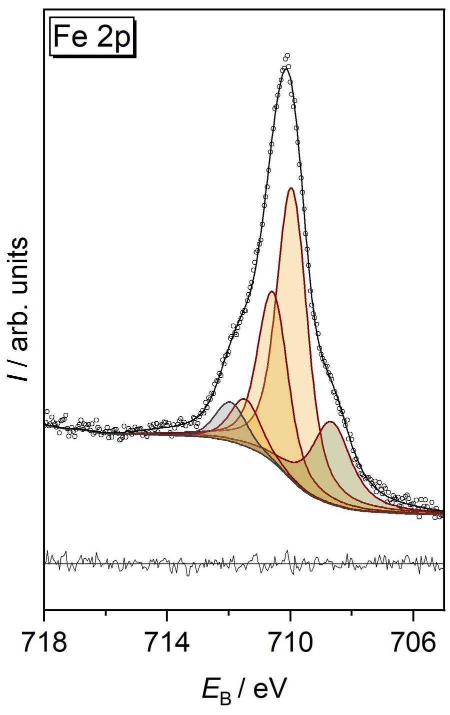

Figure S5. High-resolution Fe $2p_{3/2}$ XP spectrum of Ni-HCF nanoparticles.

Table S9. Fitted peaks of Fe2p$_{3/2}$ high-resolution XP spectrum of Ni-HCF nanoparticles.

E_B[eV]	Assignment	Reference
Fe 2p$_{3/2}$		
708.7	low-spin Fe^{2+}	[4,14]
710.0	low-spin Fe^{3+}	[3,14]
710.6	low-spin Fe^{3+}	[3,14]
711.5	low-spinFe^{3+}	[3,14]
711.9	Satellite	[14]

SI-3 XRD Measurements

Grazing incidence X-ray diffraction (GI-XRD) was performed with a PANalytical Empyrean diffractometer (Malvern Panalytical Ltd, Malvern, UK) using Cu K$_\alpha$ (λ = 1.54 Å). The angle of incidence was 2° for ITO, 0.15° for Fe-HCR, 2° for Ni-HCF nanoparticles, 0.5° for the layered material and 0.7° for the mixed material.

Figure S6 shows the XRD patterns of Fe-HCR, Ni-HCF, the layered material and the mixed material. Both, Fe-HCR and Ni-HCF materials are known to have a cubic crystal structure.[16,17] The main peaks can be assigned to the (200), (220) and (400) reflections of the materials. It is noteworthy to mention that ITO patterns overlay the materials patterns, even though the intensities are negligible in the (200) and (220) crystal plane regions. Patterns from Fe-HCR occur at lower angles compared to those of Ni-HCF due to the slightly larger unit cell of Fe-HCR.

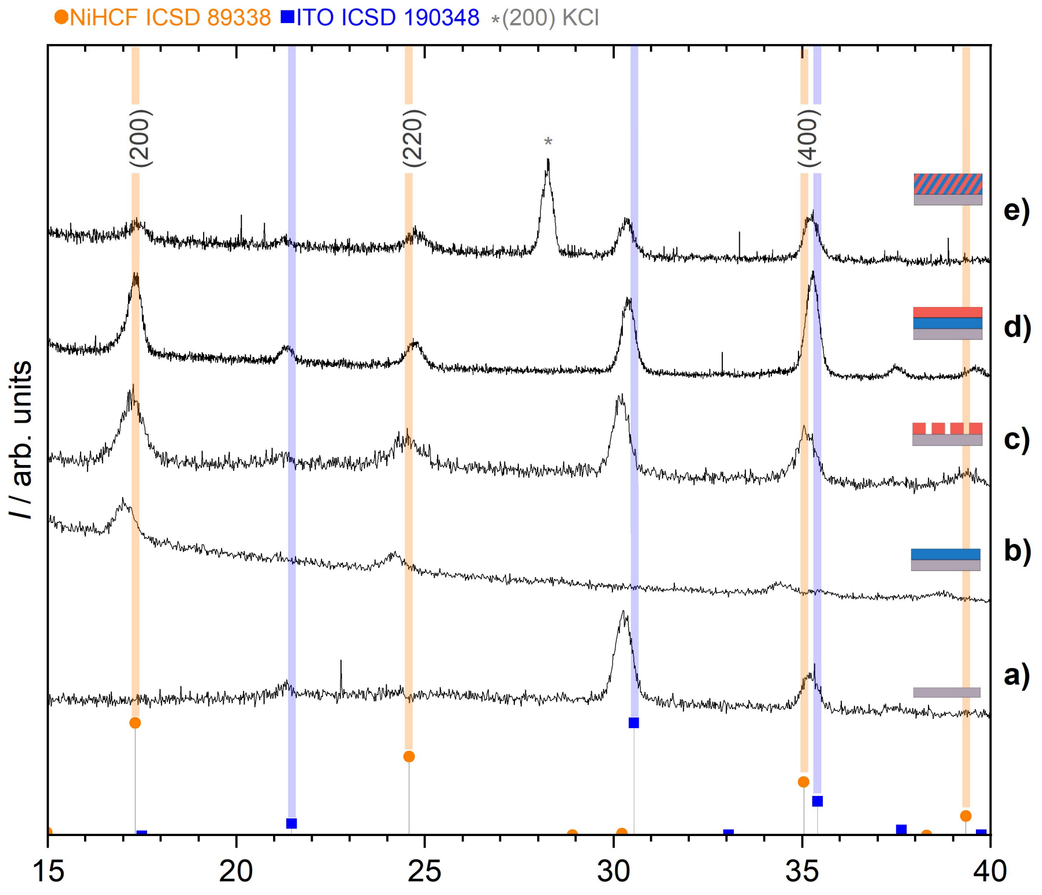

Figure S6. Grazing Incidence X-ray diffraction (GI-XRD) patterns of a) ITO; b) Fe-HCR thin film; c) Ni-HCF nanoparticles; d) layered material of Fe-HCR and Ni-HCF; e) mixed material of Fe-HCR and Ni-HCF. Symbols indicate position of reflections according to literature.

SI-4 SFM Measurements

SFM images in Figure S7a indicate that Fe-HCR forms a continuous film that is composed of small crystals. The image roughness (root mean-square roughness R_q on 10 µm × 10 µm) is 32 nm (Table 10). The film thickness is about 80 nm.

Figure S7b shows the images of the layered material of Fe-HCR as inner layer and Ni-HCF as outer layer. Ni-HCF grows in larger crystals compared to the Fe-HCR film, the roughness R_q is 21 nm on 10 µm × 10 µm. The film thickness of the layered sample is 190 nm. With the thickness of the layered material and the thickness of the Fe-HCR film, the approximate thickness of Ni-HCF nanoparticles can be determined as 190 nm – 80 nm = 110 nm.

Figure S7c shows Ni-HCF nanoparticles on the Fe-HCR film. The analysis of their height as difference between z-coordinate on top of the particle vs the z-coordinate on the Fe-HCR film yields a mean value of 120 nm ($N = 12$).

SFM images of the mixed sample (Figure S7d) show a rougher film compared to the other samples of $R_q = 45$ nm on 10 µm × 10 µm (Table S10). The increased roughness can result from alternating electrochemical deposition with different average amounts of Fe-HCR or Ni-HCF being deposited in one cycle.

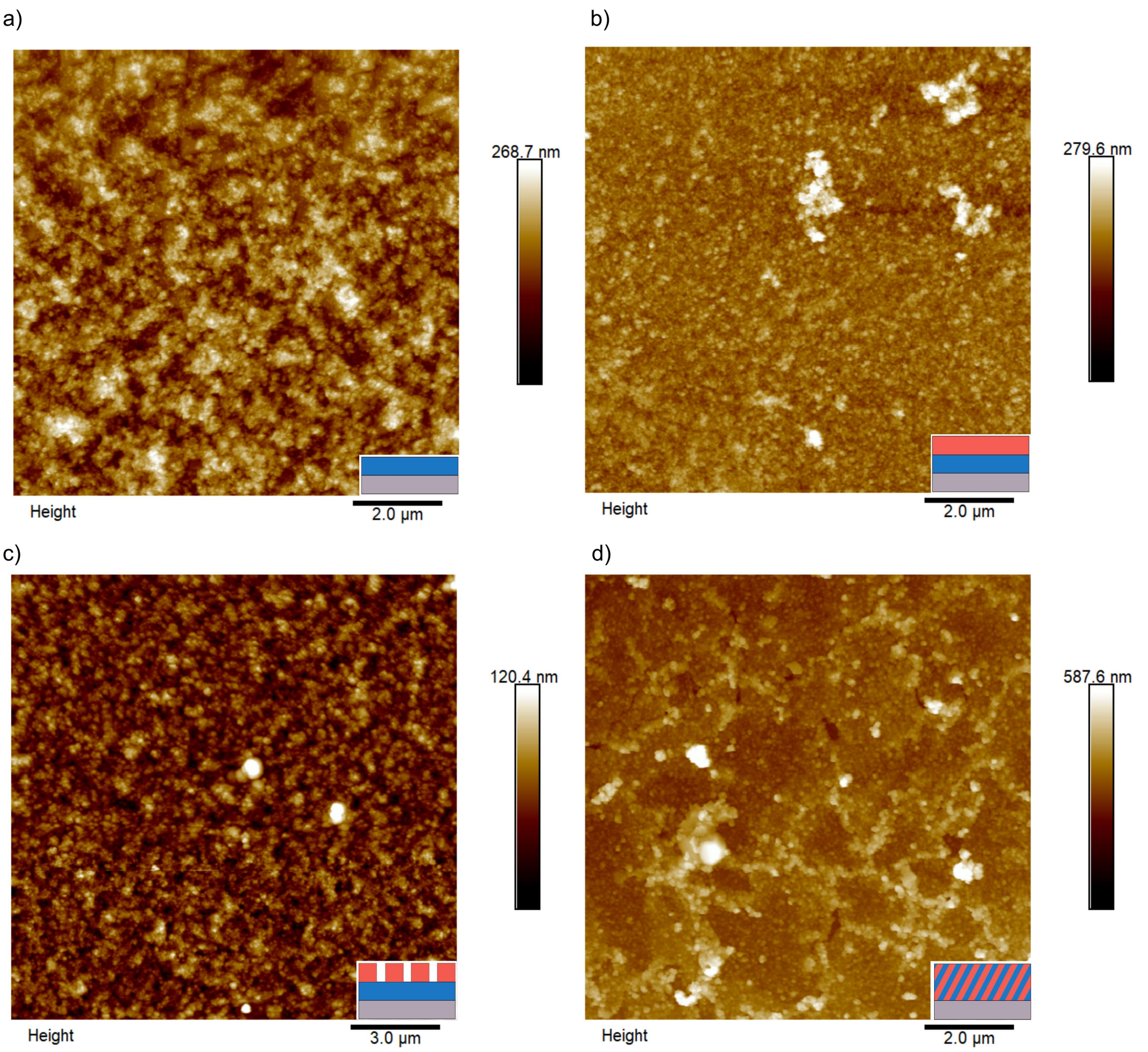

Figure S7. SFM measurements of a) Fe-HCR, 10 µm × 10 µm; b) layered material, 10 µm × 10 µm; c) Ni-HCF nanoparticles on Fe-HCR thin film, 15 µm × 15 µm; d) mixed material in 10 µm × 10 µm.

Table S10. Root mean square roughness R_q measured by SFM on 10 µm × 10 µm.

Sample	Roughness (R_q) / nm	Film thickness / nm
Fe-HCR	32	80
Fe-HCR / NiHCF layered	21	190
Fe-HCR / NiHCF layered, Ni-HCF layer	-	110 = 190-80
Fe-HCR / Ni-HCF mixed	45	240

SI-5 TEM Measurements of Ni-HCF Nanoparticles

Ni-HCF nanoparticles were synthesized according to the procedure of Li et al.[18] Briefly, a 100 mL aqueous solution of 80 mmol L^{-1} $NiCl_2 \times 6\ H_2O$ and an equal volume of 73.5 mmol L^{-1} $K_3[Fe(CN)_6]$ were mixed by simultaneously dropwise addition (10 mL h^{-1} of each solution) to 200 mL of deionized water. After complete addition, the solution was stirred for 18 h. The nanoparticles were centrifuged four times for 15 min at 4200 rpm (Megafuge 16, Thermo Scientific, USA), and stored in aqueous solution in a fridge at 4 °C for further use. The procedure is a controlled precipitation synthesis which results in nanoparticles of cubic shape. The nanoparticles on a Fe-HCR film serve as model system for the electronically communicating layered material, therefore no capping agent was used in the synthesis of the Ni-HCF nanoparticles.

Transmission electron microscopy (TEM, Zeiss EM 900N, Carl Zeiss AG, Oberkochen, Germany) was performed with an acceleration voltage of 80 kV. 8 µL of nanoparticle suspension was dropcasted on a copper grid with formvar film (300 mesh, Plano GmbH, Wetzlar, Germany). The mean diameter of the nanoparticles was calculated from 469 particles using ImageJ software.[19,20]

Ni-HCF nanoparticles of cubic shape can be found with most of the nanoparticles having a size of between 170 to 250 nm (Figure S8).

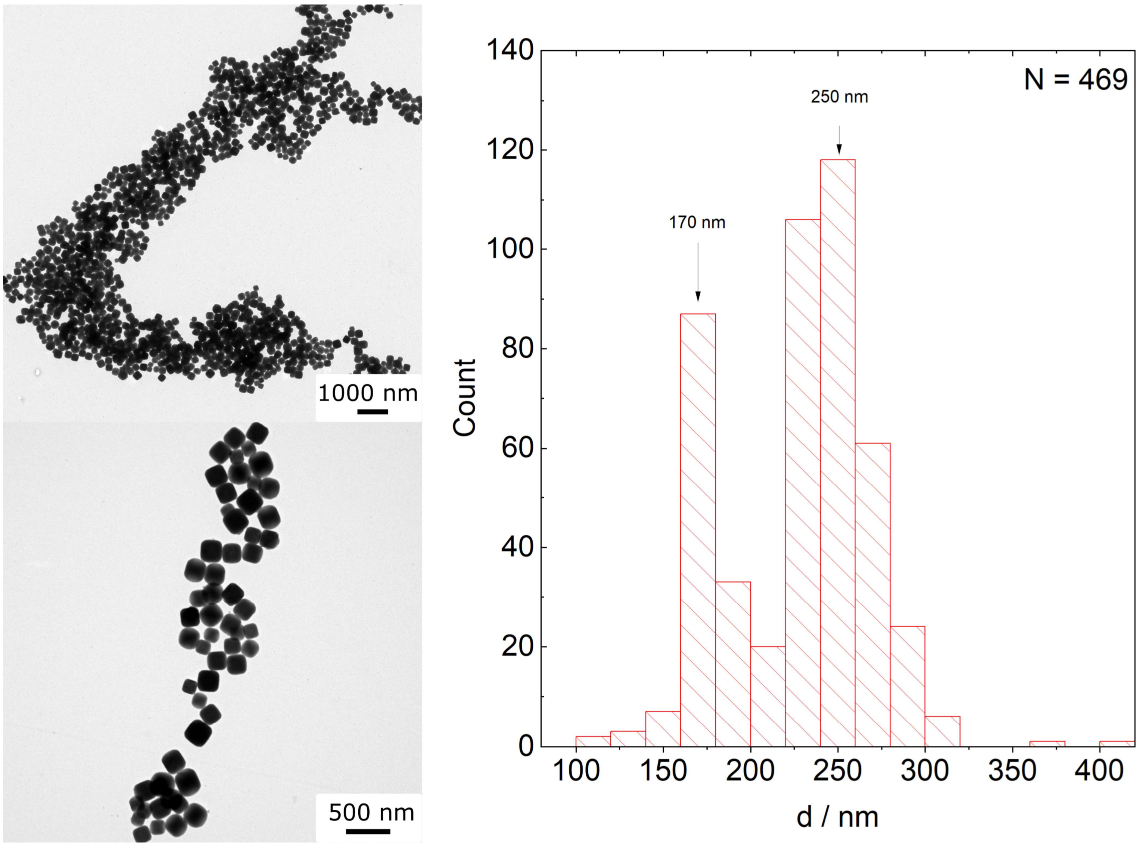

Figure S8. Transmission electron microscopy of Ni-HCF nanoparticles.

SI-6 Recording of *I-V* Curves

SI-6.1 Experimental Procedure

The *I-V* curves were recorded with conductive doped-diamond tips (CDT-FMR, Nano and More, Wetzlar, Germany, tip radius 100-200 nm according to supplier specifications) attached to a SFM stage (Enviroscope, Veeco Instruments Inc., Santa Barbara, CA, USA) controlled by a Nanoscope IIIa controller (Veeco). The application of the potential and the measurement of the current was carried out with the instrument controller. The measured current showed an offset, which was quantified by taking the current reading when the tips were placed on a microscope glass slide. This offset current was subtracted from all *I-V* curves shown in Figure 5 of the main text.

I-V curves were recorded on different regions of each sample. Figure 5 of the main text shows the individual *I-V* curves (in gray) and the average curve obtained from each sample as a thick solid line.

Table S11. Number of replicate *I-V* curves recorded over each sample and shown in Figure 5 of the main text.

Sample	Number of measurements
Fe-HCR (Figure 5a)	4
Fe-HCR|Ni-HCF nanoparticles (Figure 5b)	10
Mixed material (Figure 5c)	110
Fe-HCR|NiHCF (Figure 5d)	100

SI-6.2 Details of *I-V* Curves

The overlayed curves in Figure 5 of the main manuscript demonstrate the variability of *I-V* curves at different locations of the sample, but do not show characteristic features of individual curves with sufficient details. Therefore, representative curves from Figure 5c (mixed material) and Figure 5d (layered material) are shown below with expanded scale in Figure S9 and S10, respectively.

The curves from the layered material in Figure S10 have similar shapes. The specific currents vary as expected from slightly different contact areas of the probe. The examples from the

mixed materials in Figure S9 show also qualitative differences. Curves 1 and 2 in Figure S9a, b are close to the behavior of the layered material in Figure S10. In contrast, the curves 4 and especially curve 3 are more resembling an ohmic behavior.

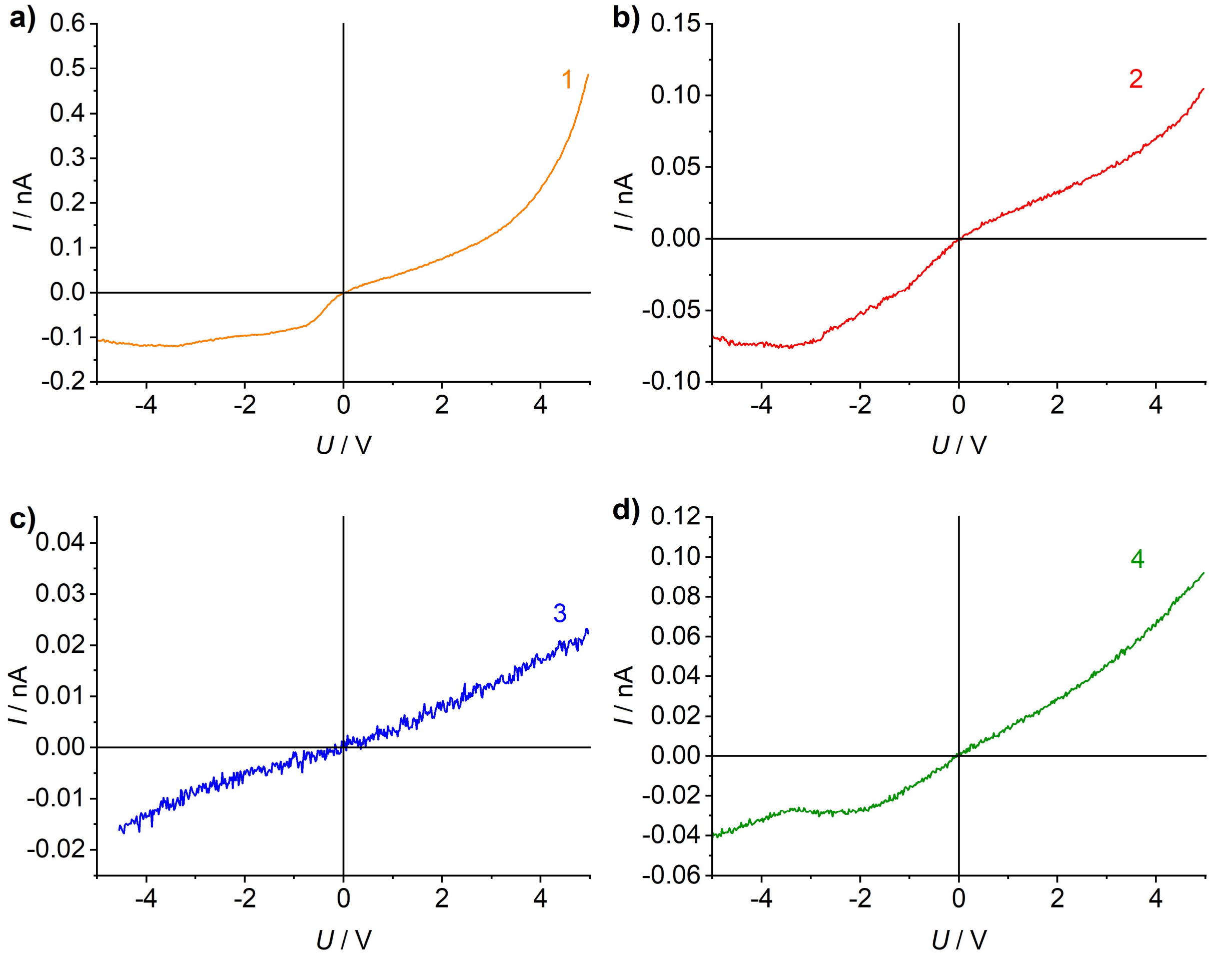

Figure S9. a)-d) Selected *I*-*V* curves of the mixed material from Figure 5c of the main text. The numbers of the curves correspond to those in Figure 5c.

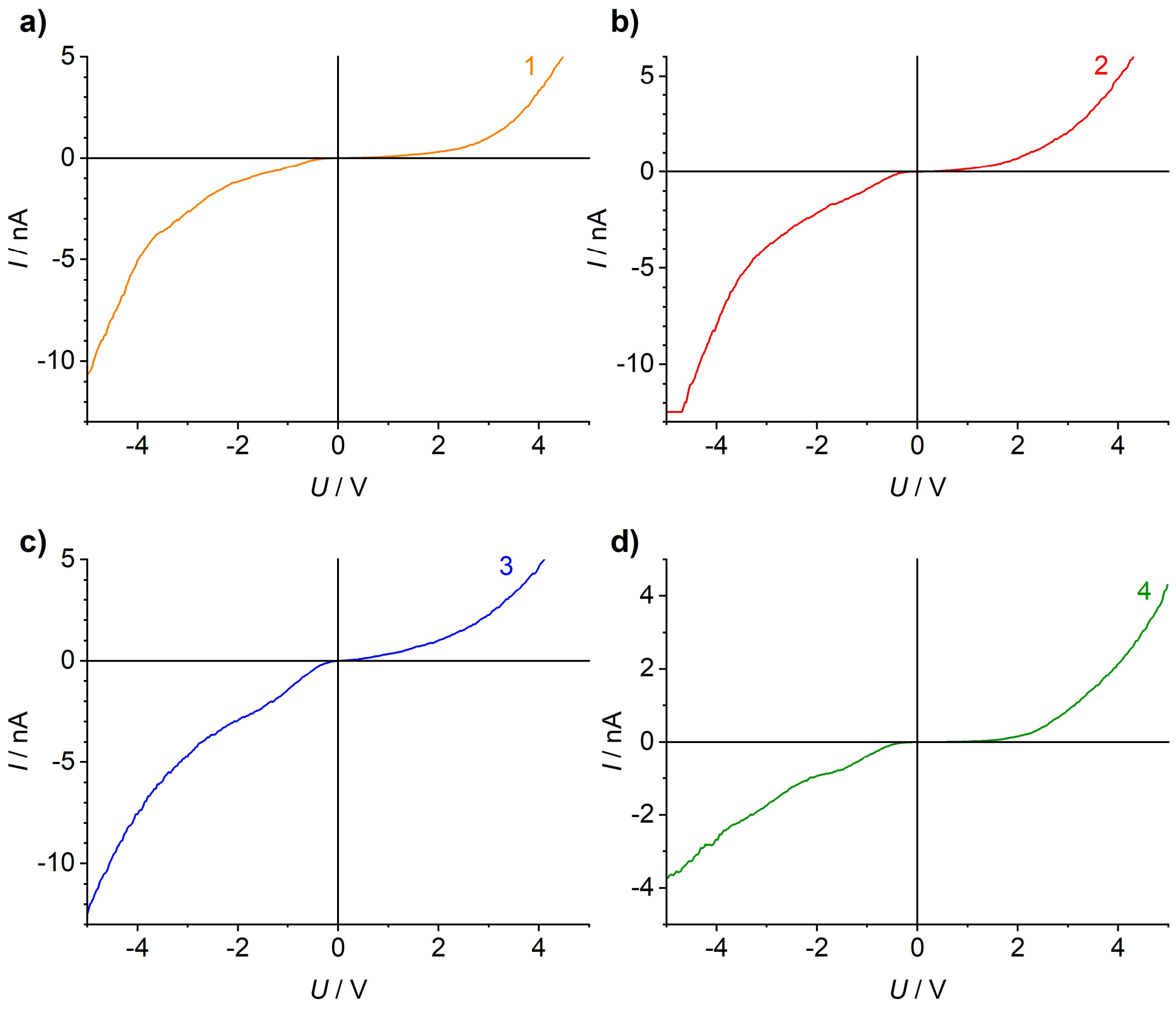

Figure S10. a)-d) Selected *I-V* curves of the layered material from Figure 5d of the main text. The numbers of the curves correspond to those in Figure 5d.

SI-6.3 Comparison of Currents in I-V curves on Fe-HCR and Fe-HCR|Ni-HCF

The currents in the *I-V* curves of a layer of Fe-HCR (Figure 5a of the main manuscript) are significantly lower than those of the layered Fe-HCR|Ni-HCR film (Figure 5d) although the same tips and experimental parameters were used. We assume that that this is a result of the different film morphologies. Electrodeposited Fe-HCR has a pronounced granular structure that is shown in Figure 2a. This structure is typical for electrodeposited films. The granular structure makes it likely, that the current is conducted through a limited number of grains as schematically depicted in Figure 11a, because gaps between grains and grain boundaries are likely to have a much higher resistance than the material itself.

In contrast, the Ni-HCF layer in the layered structure Fe-HCR|Ni-HCF was obtained by the layer-by-layer procedure and has a distinctly different morphology (Figure 2b). This may be caused by the different growth mechanisms. The smoother appearance of the film implies less grain boundaries. Usually the surface roughness decreases compared to the underlaying Fe-HCR film. As such the Ni-HCF may contact several grains of the underlaying Fe-HCR film and enables the distribution over the current over more grains than can be assessed by a direct contact of the conducting SFM tip (Figure 11b).

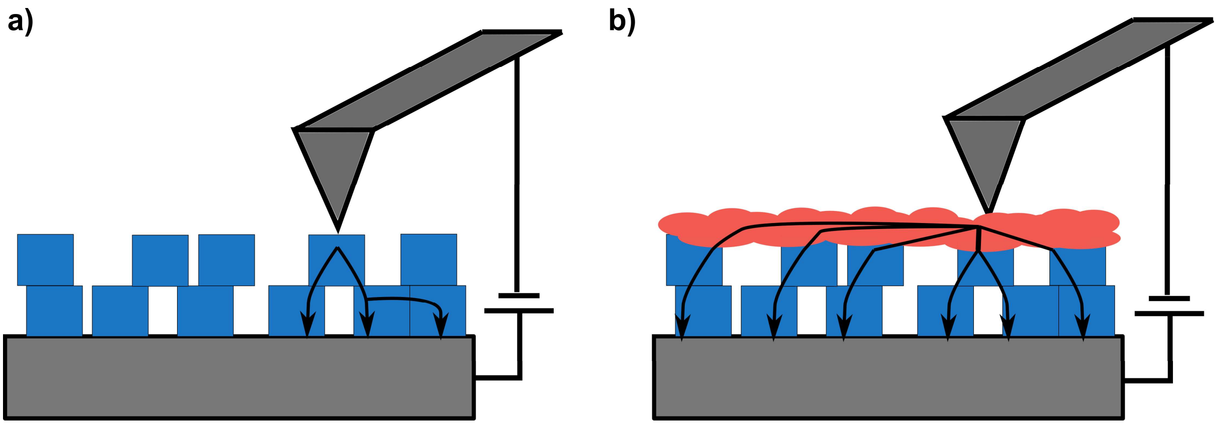

Figure S11. Schematic illustration of possible current path in I-V measurements in a) electrodeposited Fe-HCR and b) Ni-HCF layer prepared by layer-by-layer deposition on top of Fe-HCR.

SI-7 In-situ Polarization Modulation Infrared Reflection Absorption Spectroscopy

SI-7.1 Experimental Details

Polarization modulation infrared reflection absorption spectroscopy (PM IRRAS) was performed using a Vertex 70 spectrometer with an external reflection unit built around a polarization modulator (PMA 50). The PM IRRA spectra were processed using the OPUS v5.5 software. All hardware and software components were from Bruker (Ettlingen, Germany). All spectra were recorded in an in-house build spectroelectrochemical cell composed of CaF_2 optical window, H_2O electrolyte solution and metal hexacyanometallate-modified polycrystalline gold disk electrode serving as the working electrode and mirror for the IR radiation. The spectroelectrochemical cell was completed by a platinum auxiliary electrode and an Ag|AgCl|3 mol L^{-1} KCl reference electrode. All potentials are reported with respect to this reference electrode. The maximum efficiency of the photoelastic modulation was set to $\tilde{\nu}$ = 2100 cm^{-1} for the analysis of the ν(C≡N) absorption modes in hexacyanometallates films. The angle of incidence of the IR light was φ = 60° vs. the surface normal.

Before assembly of the spectroelectrochemical cell, the prism was rinsed with water, ethanol and cleaned for 10 min in an ozone chamber (Bioforce Nanosciences, Ames, USA). The cell was filled with 1 mol L^{-1} KCl electrolyte solution and purged with Ar for 30 min to remove dissolved oxygen. In each experiment positive and negative going potential scans were recorded. At selected potential, the scan was interrupted, and 100 spectra were accumulated and averaged.

SI-7.2 Fe-HCR

Spectroelectrochemical PM IRRAS data for a thin Fe-HCR film are shown in Figure S12. Fe-HCR is a solid solution of the ‘soluble’ and ‘insoluble’ structure with vacancies on the M^2 site,[21] they can cause different signals in PM IRRA spectra even for one material.[22] In the PM IRRA spectra are dominated by a strong ν(C≡N) absorption mode at ~2080 cm^{-1}. This mode

disappears in the potential range of 0.38 V – 0.65 V in the forward scan and 0.65 – 0.35 V in the back scan. Instead, a new *v*(C≡N) absorption mode appears at ~2106 cm^{-1}. In accordance with literature of other metal hexacyanometallates with redox reactions at high-spin iron, the IR absorption mode at around 2080 cm^{-1} can be assigned to Fe^{2+}-N≡C-Ru^{2+} and the IR absorption mode at ~2106 cm^{-1} can be attributed to Fe^{3+}-N≡C-Ru^{2+}.[14] The PM IRRA spectra of the oxidized material in Figure S12 still contain a shoulder at wavenumbers attributed to the reduced form. The position of this signal was found as local minimum of the second derivative of the spectra at 2042 cm^{-1} and 2056 cm^{-1} (Figure S13a). The vibration mode can be attributed to defects in the structure, like vacancies, changes in binding motif to Ru-N≡C-Fe, but also terminal cyanides.[23,22]

The assignment of the signals for the oxidized and reduced form of Fe-HCR are compiled in Table S12.

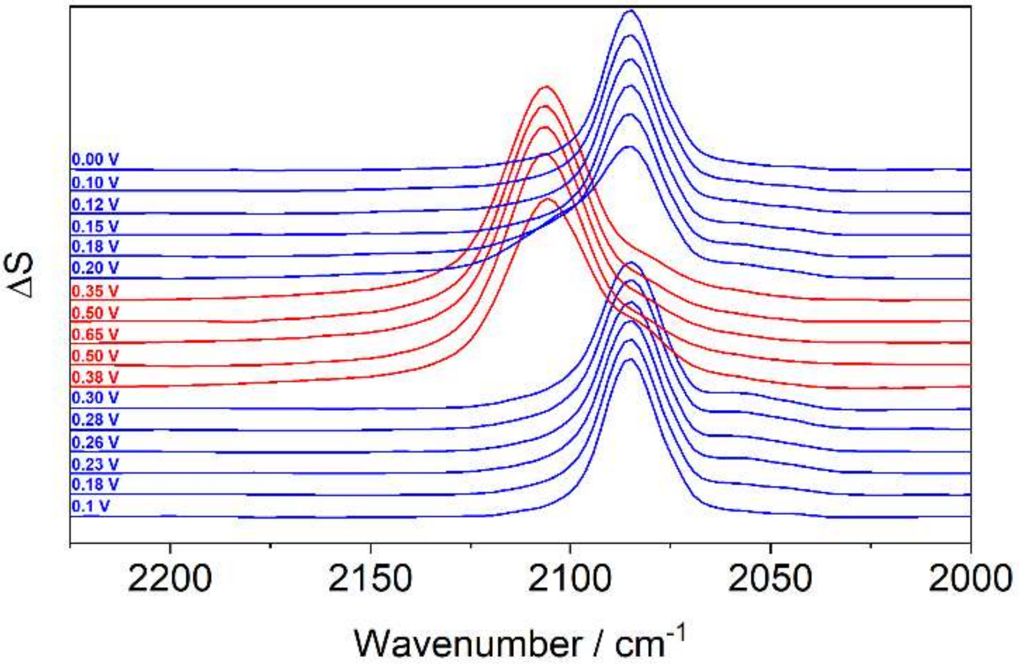

Figure S12. PM IRRA spectra in 2225- 2000 cm^{-1} region of Fe-HCR in positively and negatively potential scans.

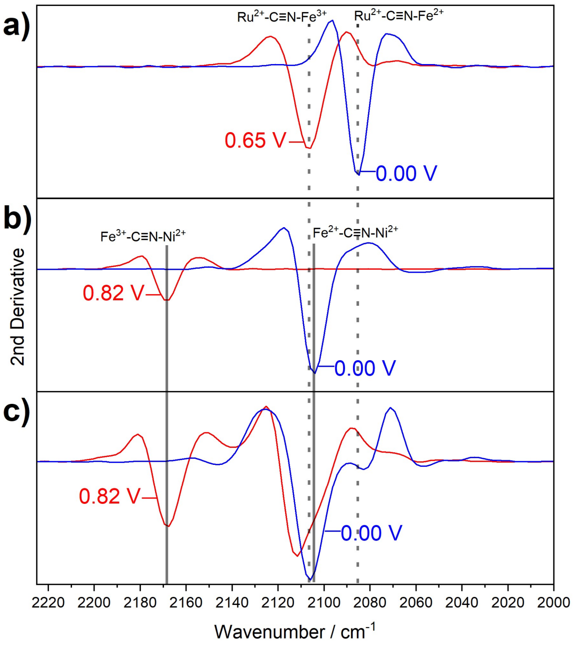

Figure S13. Second derivative of the reduced and oxidized state of the PMIRRA spectra of a) Fe-HCR, b) Ni-HCF and c) the Fe-HCR | Ni-HCF layered material.

Table S12. Wavenumber, intensity and assignment of the *ν*(C≡N) stretching mode in Fe-HCR thin films deposited on a Au electrode surface.

ν(C≡N)/cm^{-1}	Mode Intensity[a]	Assignment	References
2042	v.w.	Defects	[23,22]
2056-2057	v.w.	Defects	[23,22]
2079-2085	v.s.	Ru^{2+}-C≡N-Fe^{2+}	[14]
2106-2107	v.s.	Ru^{2+}-C≡N-Fe^{3+}	[14]

[a] v.w. – very weak; v.s. – very strong

SI-7.3 Ni-HCF

Spectroelectrochemical PM IRRAS data of a thin Ni-HCF film are shown in Figure S14. Electrochemical responses of the pure Ni-HCF thin film (CVs in Figure 4b and 4e of the main manuscript) indicate the presence of non-stoichiometric forms. The defects associated with the non-stoichiometric structure (solid solution of $K_2Ni[Fe(CN)_6]$ and $KNi_{1.5}[Fe(CN)_6]$) may have an influence on the PM IRRA spectra. In the PM IRRA spectra a strong *ν*(C≡N) absorption mode at ~2100 cm^{-1} is present. It disappears completely in the potential range of $E > 0.73$ V. Additionally, a new, rather weak absorption mode appears at 2163 cm^{-1} at potentials of ~ $E >$ 0.55 V. In accordance with literature, the strong IR absorption mode at around ~2100 cm^{-1} can

be assigned to Ni^{2+}-N≡C-Fe^{2+}. At oxidizing potentials, the IR absorption mode at ~ 2163 cm^{-1} can be attributed to Ni^{2+}-N≡C-Fe^{3+}.[14,23–25] Beside the main features, the second derivative of the spectra (not shown) revealed further, very weak, absorption modes at ~2050 cm^{-1}, ~2065 cm^{-1}, ~2125 cm^{-1} and ~2140 cm^{-1}, which can be attributed to defects in the structure and / or also terminal cyanides.[23,22] During the potential change, the intensities of the absorption modes at 2050 cm^{-1} and 2125 cm^{-1} change concomitantly. The same is observed for the modes at 2065 cm^{-1} and 2140 cm^{-1}. This allows their assignment to the oxidized and reduced forms in Table S13. In the negatively going potential scan, the absorption mode at around 2163 cm^{-1} assigned to Fe^{3+}-C≡N-Ni^{2+} disappears at potentials of $E < 0.55$ V.

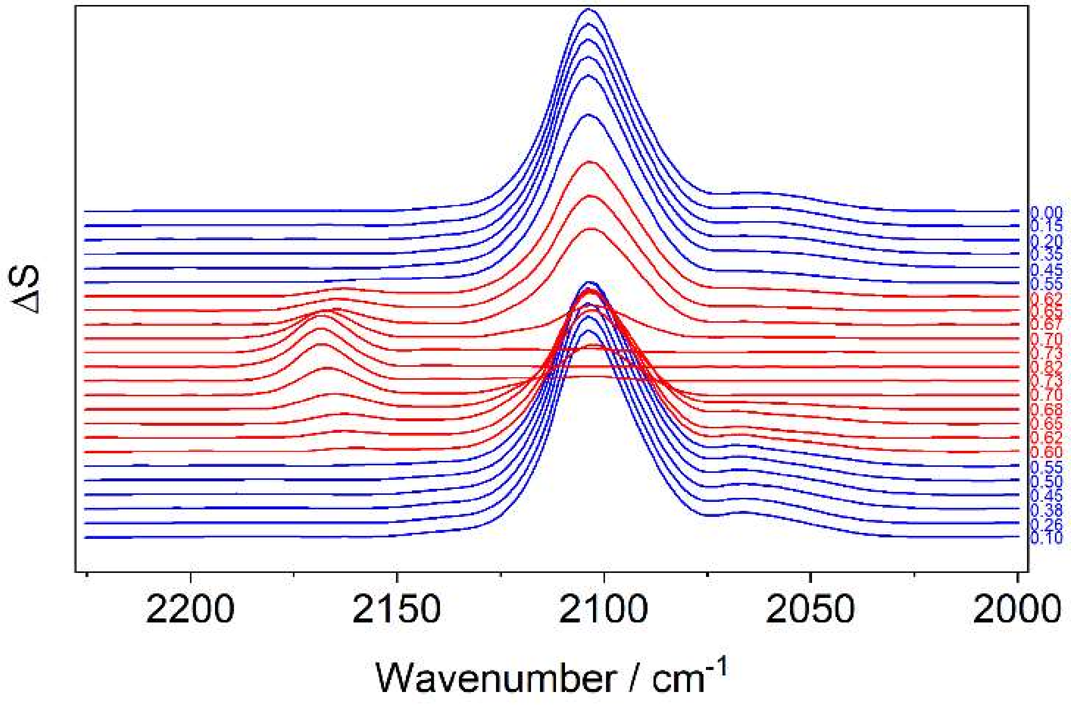

Figure S14. PM IRRA spectra in 2225- 2000 cm^{-1} region of Ni-HCF in positively and negatively potential scans.

Table S13. Wavenumber, intensity and assignment of the v(C≡N) stretching mode in Ni-HCF thin films deposited on a Au electrode surface.

v(C≡N)/cm^{-1}	Mode Intensity[a]	Assignment	References
2048-2053	v.w.	Defects	[23,22]
2063-2065	w.	Defects	[23,22]
2100-2104	v.s.	Fe^{2+}-C≡N-Ni^{2+}	[14,24,23,25]
2124-2125	v.w.	Defects	[23,22]
2140-2141	v.w.	Defects	[23,22]
2163-2169	s.	Fe^{3+}-C≡N-Ni^{2+}	[14,25,23]

[a] v.w. – very weak; w. – weak; s. – strong; v.s. – very strong

SI-7.4 Fe-HCR|Ni-HCF Layered Material

The spectroelectrochemical PM IRRAS data of the layered Fe-HCR|Ni-HCF material is shown in Figure S15. Due to overlapping peaks of two compounds and different oxidation states, leads to overlapping absorption modes of oxidized form of Fe-HCR and reduced form of Ni-HCF leading to difficulties in a precise assignment (Table S14). Signals identified from the second derivative of the spectra (Figure S13c) are shown in Table S14. Further weak absorption modes can be found in in Figure S15 at 2043 cm^{-1}, 2057 cm^{-1} and ~2140cm^{-1}. They may be assigned to defects in the structure.[22,23] The absorption mode for Fe^{2+}-C≡N-Ru^{2+} appears at 2082 cm^{-1}, the strong absorption mode for Fe^{2+}-C≡N-Ni^{2+} appears at 2105 cm^{-1}.[14,23–25] When Fe-HCR is oxidized at $E > 0.30$ V, the strong absorption mode shifts to 2111 cm^{-1}. With ongoing oxidation of the system, this peak shifts slightly more to 2114 cm^{-1}. In this wavenumber range, overlap of different absorption modes is expected. The absorption modes of Fe^{2+}-C≡N-Ni^{2+} occurs here as well as the absorption mode of Ru^{2+}-C≡N-Fe^{3+}. Indeed, the shift of the peak at potentials ($E > 0.30$ V) shows the influence of oxidation of Ru^{2+}-C≡N-Fe^{3+} on this absorption mode. Moreover, the peak intensity decreases with increasing potential indicating the oxidation of Fe^{2+}-C≡N-Ni^{2+}. The resulting oxidized form Fe^{3+}-C≡N-Ni^{2+} is associated with the absorption mode at about 2165 cm^{-1}. This absorption mode can be found in the PM IRRA spectra at around 0.45 V (Figure S15).[14,23,25] In the negatively going scan, the absorption mode at around 2165 cm^{-1} (assigned to Fe^{3+}-C≡N-Ni^{2+}) disappears at potentials of $E < 0.30$ V.

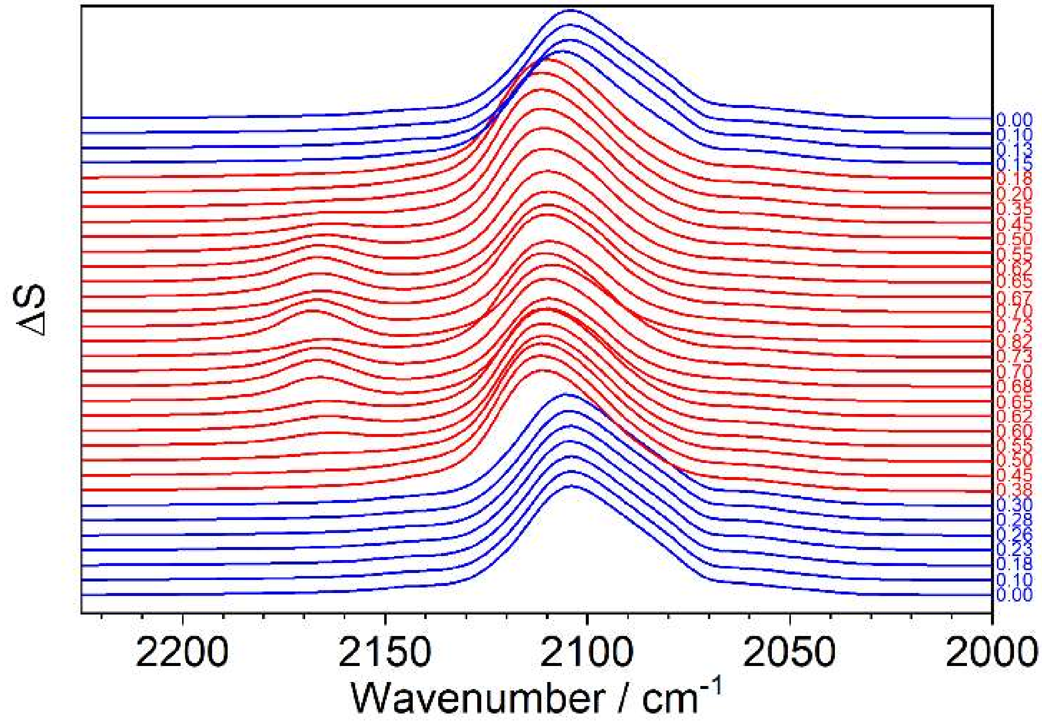

Figure S15. PM IRRA spectra in 2225- 2000 cm^{-1} region of Fe-HCR|Ni-HCF layered material in positive and negative potential scans.

Table S14. Wavenumber, intensity and assignment of the v(C≡N) stretching mode in Fe-HCR/Ni-HCF layered material thin films deposited on an Au electrode surface.

***v*(C≡N)/cm^{-1}**	**Mode Intensity[a]**	**Assignment**	**References**
2043	v.w.	Defects	[22,23]
2057	w	Defects	[22,23]
2082	m.s.	Ru^{2+}-C≡N-Fe^{2+}	[14]
2105	v.s.	Fe^{2+}-C≡N-Ni^{2+}	[14,23–25]
2111-2114	v.s.	Fe^{2+}-C≡N-Ni^{2+} / Ru^{2+}-C≡N-Fe^{3+}	[14]
2138-2145	w	Defects	[22,23]
2164-2167	s	Fe^{3+}-C≡N-Ni^{2+}	[14,23,25]

[a] v.w. – very weak; w. – weak; m.s. – medium strong; s. – strong; v.s. – very strong

SI-8 Attempts on Other Layered Systems

It is conceivable that alternative combination of metal hexacyanometallate layers can also promote unidirectional current flow and charge trapping provided the redox potentials exhibit a significant difference. However, there must be also a structural compatibility, if the films are grown on top of each other without intermediate organic films or the use of binders.

SI-8.1 Fe-HCR | Cu-HCF

The bilayer system Fe-HCR | Cu-HCF shows a potential difference that could be suitable to achieve rectifying behavior and charge trapping.

The inner Fe-HCR film was prepared as described in the main text. The outer Cu-HCF layer was deposited by layer-by-layer (LbL) deposition aqueous 20 mmol L^{-1} $CuCl_2$ (Merck) and aqueous 20 mmol L^{-1} $K_3[Fe(CN)_6]$ (Alfa Aesar) solution. The samples were exposed to each solution for 20 min, followed by 30 s rinsing with water. In total, 18 cycles were performed.

I-V curves recorded with a conductive scanning force microscopy on a Cu-HCF thin film deposited on Fe-HCR demonstrated an asymmetrical response (Figure S16). However, the currents are in the range that is also measured for an Fe-HCR film (Figure 5a). Thus the Cu-HCF most likely cannot electrically connect the different grains of the Fe-HCR film as the Ni-HCF film presented in the main text. The extent of asymmetry in the *I-V* curves is also less pronounced than in the layered Fe-HCR|Ni-HCF film (Figure 5d).

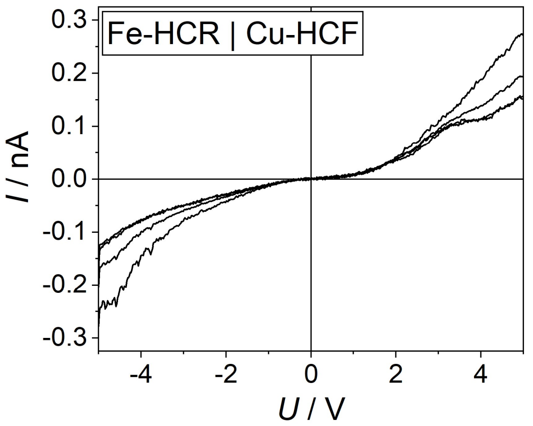

Figure S16. *I-V* curves on such films recorded between the support electrode and an C-SFM tip on a layered Fe-HCR|Cu-HCF structure.

SI-8.2 Systems with Zn-HCF

Due to the distinctly different redox potential and the contained abundant elements, Zn-HCF was also tested as component of layered architectures.

Electrodeposition of thin Zn-HCF was performed on a cleaned ITO surface in a solution of 0.1 mol L^{-1} $ZnCl_2$ (Sigma Aldrich) + 0.05 mol L^{-1} $K_3[Fe(CN)_6]$ + 0.5 mol L^{-1} KCl + 0.1 mol L^{-1} HCl in a potential window of 0 to 1.2 V at v = 50 mV s^{-1} (Figure S17).

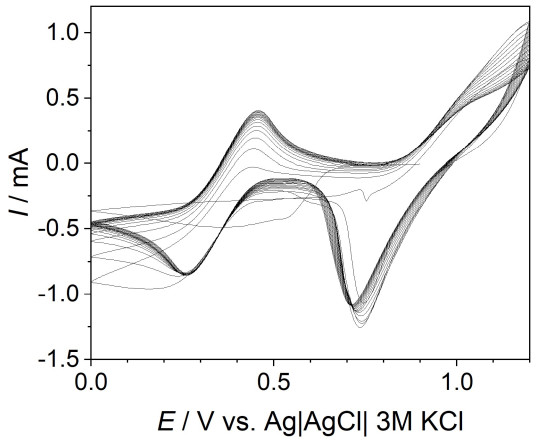

Figure S17. Electrochemical deposition of Zn-HCF in 0.1 mol L^{-1} $ZnCl_2$ + 0.05 mol L^{-1} $K_3Fe(CN)_6$ + 0.5 mol L^{-1} KCl + 0.1 mol L^{-1} HCl; v = 0.04 Vs^{-1}.

Electrodeposited films of Zn-HCF does not show a uniform film morphology (Figure S18). As depicted in Figure S17, the electrodeposition of Zn-HCF necessitates higher potentials than compatible with aqueous electrolyte solutions and gold electrodes. Au electrodes are preferred here because they are more suitable for subsequent XPS characterization. Electrodes from thin conducting oxides (TCO) may be suitable for the electrodeposition and electrochemical characterization of Zn-HCF, but the Sn 3p photoemission signal overlaps with the Fe 2p signals and makes a detailed XP analysis impossible. Furthermore, the oxide component of TCOs also interferes with the O 1s signal in XPS.

An attempt to prepare Ni-HCF|Zn-HCF as a layered system by electrodeposition of Ni-HCF followed by LbL deposition of Zn-HCF was not successful because the material detached during the LbL process. This highlights restrictions imposed by material compatibility.

Procedures: Ni-HCF was electrochemically deposited during 15 potential cycles at v = 40 mV s^{-1} between -0.0 and 0.75 V in 1 mmol L^{-1} $NiCl_2$ + 0.5 mmol L^{-1} $K_3[Fe(CN)_6]$ + 500 mmol L^{-1} KCl. Zn-HCF was deposited in 10 cycles from aqueous solution of 20 mmol L^{-1} $ZnCl_2$ and 20 mmol L^{-1} $K_3[Fe(CN)_6]$ by LbL. The samples were rinsed with water for 30 s in between the immersion in the two precursor solutions.

a) b)

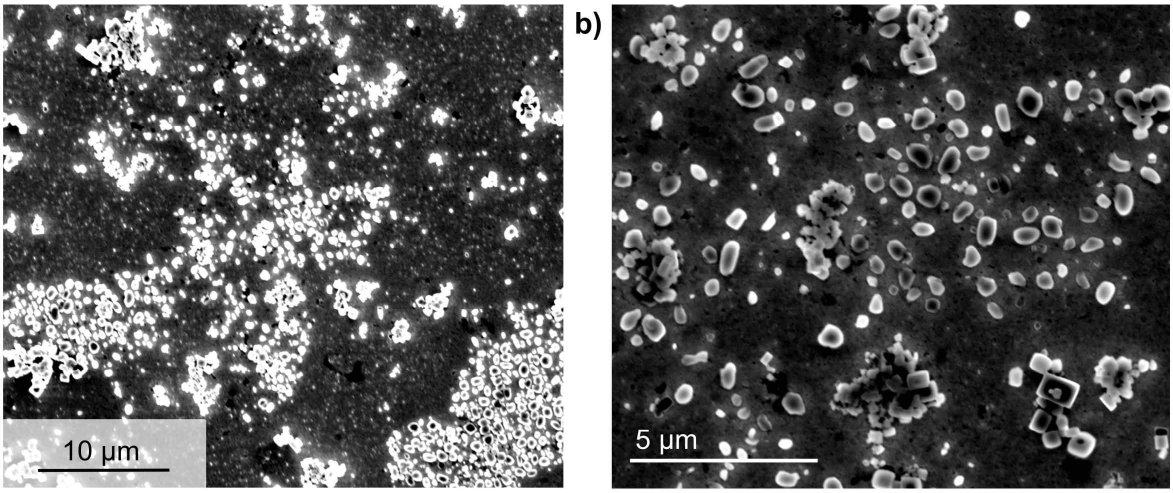

Figure S18. SEM images of a Zn-HCF thin film prepared by electrochemical deposition at different locations of the film.

SI-8.3 Fe-HCF|Ni-HCF

The classical system Fe-HCF|Ni-HCF used in ref 26 did not shows charge trapping when prepared by a combination of electrodeposition and layer-by-layer deposition. The systemwa prepared by electrodeposition of Fe-HCF with 0.002 mol l^{-1} $K_3[Fe(CN)_6]$ + 0.002 mol l^{-1} $FeCl_3$ (Sigma Aldrich) + 0.1 mol l^{-1} KCl + 0.1 mol l^{-1} HCl. The outer layer of Ni-HCF was deposited by LbL deposition as described in the main text.

Figure S19 shows the obtained cyclic voltammogram of the layered Fe-HCF|Ni-HCF system, which is simply a superposition of the signals for Fe-HCF and Ni-HCF. The system did not show charge trapping when prepared by a combination of electrodeposition and layer-by-layer deposition. This is similar to the result obtained in ref 27 by combining two electrodeposition steps (without binder).

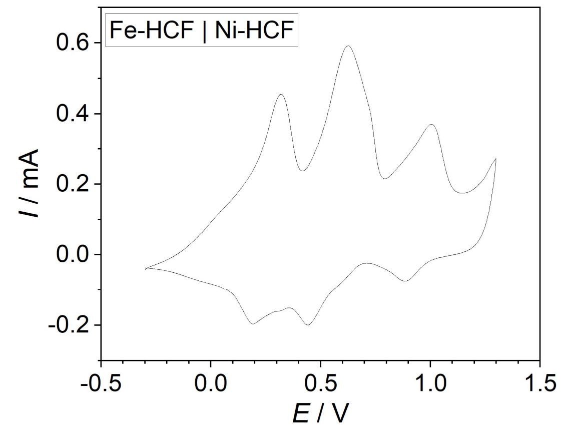

Figure S19. Cyclic voltammogram of layered Fe-HCF|Ni-HCF system; v = 0.01 Vs^{-1}.

References

(1) Wang, Y.; Rui, Y.; Li, F.; Li, M. Electrodeposition of Nickel Hexacyanoferrate/layered Double Hydroxide Hybrid Film on the Gold Electrode and Its Application in the Electroanalysis of Ascorbic Acid. *Electrochim. Acta* **2014**, *117*, 398–404. https://doi.org/10.1016/j.electacta.2013.11.141.

(2) Zamponi, S.; Berrettoni, M.; Kulesza, P. J.; Miecznikowski, K.; Malik, M. A.; Makowski, O.; Marassi, R. Influence of Experimental Conditions on Electrochemical Behavior of Prussian Blue Type Nickel Hexacyanoferrate Film. *Electrochim. Acta* **2003**, *48*, 4261–4269. https://doi.org/10.1016/j.electacta.2003.08.001.

(3) Gupta, R. P.; Sen, S. K. Calculation of Multiplet Structure of Core P -Vacancy Levels. II. *Phys. Rev. B: Solid State* **1975**, *12*, 15–19. https://doi.org/10.1103/PhysRevB.12.15.

(4) Grosvenor, A. P.; Kobe, B. A.; Biesinger, M. C.; McIntyre, N. S. Investigation of Multiplet Splitting of Fe 2p XPS Spectra and Bonding in Iron Compounds. *Surf. Interface Anal.* **2004**, *36*, 1564–1574. https://doi.org/10.1002/sia.1984.

(5) Benedetto, G. E. de; Guascito, M. R.; Ciriello, R.; Cataldi, T. R.I. Analysis by X-Ray Photoelectron Spectroscopy of Ruthenium Stabilised Polynuclear Hexacyanometallate Film Electrodes. *Anal. Chim. Acta* **2000**, *410*, 143–152. https://doi.org/10.1016/S0003-2670(00)00724-8.

(6) Weidinger, D.; Brown, D. J.; Owrutsky, J. C. Transient Absorption Studies of Vibrational Relaxation and Photophysics of Prussian Blue and Ruthenium Purple Nanoparticles. *J. Chem. Phys.* **2011**, *134*, 124510. https://doi.org/10.1063/1.3564918.

(7) Sauter, S.; Wittstock, G.; Szargan, R. Localisation of Electrochemical Oxidation Processes in Nickel and Cobalt Hexacyanoferrates Investigated by Analysis of the Multiplet Patterns in X-Ray Photoelectron Spectra. *Phys. Chem. Chem. Phys.* **2001**, *3*, 562–569. https://doi.org/10.1039/b008430l.

(8) Hosseini, P.; Wolkersdörfer, K.; Wark, M.; Redel, E.; Baumgart, H.; Wittstock, G. Morphology and Conductivity of Copper Hexacyanoferrate Films. *J. Phys. Chem. C* **2020**, *124*, 16849–16859. https://doi.org/10.1021/acs.jpcc.0c06114.

(9) Malik, M. A.; Kulesza, P. J.; Wlodarczyk, R.; Wittstock, G.; Szargan, R.; Bala, H.; Galus, Z. Formation of Ultra-Thin Prussian Blue Layer on Carbon Steel that Promotes Adherence of Hybrid Polypyrrole Based Protective Coating. *J. Solid State Electrochem.* **2005**, *9*, 403–411.

(10) Cano, A.; Rodríguez-Hernández, J.; Reguera, L.; Rodríguez-Castellón, E.; Reguera, E. On the Scope of XPS as Sensor in Coordination Chemistry of Transition Metal Hexacyanometallates. *Eur. J. Inorg. Chem.* **2019**, *2019*, 1724–1732. https://doi.org/10.1002/ejic.201801556.

(11) Cano, A.; Reguera, L.; Avila, M.; Velasco-Arias, D.; Reguera, E. Charge Redistribution Effects in Hexacyanometallates Evaluated from XPS Data. *Eur. J. Inorg. Chem.* **2020**, *2020*, 137–145. https://doi.org/10.1002/ejic.201900907.

(12) Ahn, W.; Park, M. G.; Lee, D. U.; Seo, M. H.; Jiang, G.; Cano, Z. P.; Hassan, F. M.; Chen, Z. Hollow Multivoid Nanocuboids Derived from Ternary Ni–Co–Fe Prussian Blue Analog for Dual-Electrocatalysis of Oxygen and Hydrogen Evolution Reactions. *Adv. Funct. Mater.* **2018**, *28*, 1802129. https://doi.org/10.1002/adfm.201802129.

(13) Cano, A.; Lartundo-Rojas, L.; Shchukarev, A.; Reguera, E. Contribution to the Coordination Chemistry of Transition Metal Nitroprussides: A Cryo-XPS Study. *New J. Chem.* **2019**, *43*, 4835–4848. https://doi.org/10.1039/C9NJ00141G.

(14) Gerber, S. J.; Erasmus, E. Electronic Effects of Metal Hexacyanoferrates: An XPS and FTIR Study. *Mater. Chem. Phys.* **2018**, *203*, 73–81. https://doi.org/10.1016/j.matchemphys.2017.09.029.

(15) Cataldi, T. R. I.; Salvi, A. M.; Centonze, D.; Sabbatini, L. Voltammetric and XPS Investigations of Polynuclear Ruthenium-Containing Cyanometallate Film Electrodes. *J. Electroanal. Chem.* **1996**, *406*, 91–99. https://doi.org/10.1016/0022-0728(95)04426-4.

(16) Jain, V.; Sahoo, R.; Jinschek, J. R.; Montazami, R.; Yochum, H. M.; Beyer, F. L.; Kumar, A.; Heflin, J. R. High Contrast Solid State Electrochromic Devices Based on Ruthenium Purple Nanocomposites Fabricated by Layer-by-Layer Assembly. *Chem. Commun.* **2008**, 3663–3665. https://doi.org/10.1039/B803915A.

(17) Wessells, C. D.; Peddada, S. V.; Huggins, R. A.; Cui, Y. Nickel Hexacyanoferrate Nanoparticle Electrodes for Aqueous Sodium and Potassium Ion Batteries. *Nano Lett.* **2011**, *11*, 5421–5425.

(18) Li, C. H.; Nanba, Y.; Asakura, D.; Okubo, M.; Talham, D. R. Li-Ion and Na-Ion Insertion into Size-Controlled Nickel Hexacyanoferrate Nanoparticles. *RSC Adv.* **2014**, *4*, 24955–24961. https://doi.org/10.1039/C4RA03296A.

(19) Schneider, C. A.; Rasband, W. S.; Eliceiri, K. W. NIH Image to ImageJ: 25 Years of Image Analysis. *Nat. Methods* **2012**, *9*, 671–675. https://doi.org/10.1038/nmeth.2089.

(20) Rasband, W. S. *Image J.*; U. S. National Institutes of Health, Bethesda, Maryland, USA, 1997. https://imagej.nih.gov/ij/.

(21) Abe, T.; Toda, G.; Tajiri, A.; Kaneko, M. Electrochemistry of Ferric Ruthenocyanide (Ruthenium Purple), and Its Electrocatalysis for Proton Reduction. *J. Electroanal. Chem.* **2001**, *510*, 35–42. https://doi.org/10.1016/S0022-0728(01)00539-3.

(22) Hosseini, P.; Wittstock, G.; Brand, I. Infrared Spectroelectrochemical Analysis of Potential Dependent Changes in Cobalt Hexacyanoferrate and Copper Hexacyanoferrate Films on Gold Electrodes. *J. Electroanal. Chem.* **2018**, *812*, 199–206. https://doi.org/10.1016/j.jelechem.2017.12.029.

(23) Pajerowski, D. M.; Gardner, J. E.; Talham, D. R.; Meisen, M. W. Tuning the Sign of Photoinduced Changes in Magnetization: Spin Transitions in the Ternary Metal Prussian Blue Analogue NaαNi1−xCox[Fe(CN)6]B·nH2O. *J. Am. Chem. Soc.* **2009**, *131*, 12927–12936.

(24) Goberna-Ferrón, S.; Hernándes, W. Y.; Rodríguez-García, B.; Galán-Mascarós, J. R. Light-Driven Water Oxidation with Metal Hexacyanometallate Heterogeneous Catalysts. *ACS Catal.* **2014**, *4*, 1637–1641.

(25) Banavath, R.; Abhinav, A.; Srivastava, R.; Bhargava, P. Highly Sensitive Ascorbic Acid Sensors from EDTA Chelation Derived Nickel Hexacyanoferrate/ Graphene Nanocomposites. *Electrochim. Acta* **2022**, *419*, 140335.

(26) Miecznikowski, K.; Chojak, M.; Steplowska, W.; Malik, M.; Kulesza, P. Microelectrochemical Electronic Effects in Two-Layer Structures of Distinct Prussian Blue Type Metal Hexacyanoferrates. *J. Solid State Electrochem.* **2004**, *8*, 868–875. https://doi.org/10.1007/s10008-004-0555-4.

(27) Karpova, E. V.; Karyakina, E. E.; Karyakin, A. A. Iron–nickel Hexacyanoferrate Bilayer as an Advanced Electrocatalyst for H_2O_2 Reduction. *RSC Adv.* **2016**, *6*, 103328–103331. https://doi.org/10.1039/C6RA24128J.
